# Chiral Recognition of Flexible Melatonin Receptor Ligands Induced by Conformational Equilibria

**DOI:** 10.3390/molecules25184057

**Published:** 2020-09-04

**Authors:** Gian Marco Elisi, Annalida Bedini, Laura Scalvini, Caterina Carmi, Silvia Bartolucci, Valeria Lucini, Francesco Scaglione, Marco Mor, Silvia Rivara, Gilberto Spadoni

**Affiliations:** 1 Dipartimento di Scienze degli Alimenti e del Farmaco, Università degli Studi di Parma, Parco Area delle Scienze 27/A, I-43124 Parma, Italy; gianmarco.elisi@unipr.it (G.M.E.); laura.scalvini@unipr.it (L.S.); caterina.carmi@yahoo.it (C.C.); silvia.rivara@unipr.it (S.R.); 2 Dipartimento di Scienze Biomolecolari, Università degli Studi di Urbino “Carlo Bo”, Piazza Rinascimento 6, I-61029 Urbino, Italy; annalida.bedini@uniurb.it (A.B.); silvia.bartolucci@uniurb.it (S.B.); gilberto.spadoni@uniurb.it (G.S.); 3 Dipartimento di Oncologia ed Emato-Oncologia, Università degli Studi di Milano, Via Vanvitelli 32, I-20129 Milano, Italy; valeria.lucini@unimi.it (V.L.); francesco.scaglione@unimi.it (F.S.)

**Keywords:** melatonin, chiral recognition, conformational analysis, molecular dynamics, stereoselectivity, UCM793

## Abstract

*N*-anilinoethylamides are a class of melatoninergic agents with the aniline portion mimicking the indole ring of the natural ligand and the ethylamide chain reproducing that of melatonin. The simplest compound in this class, *N*-{2-[(3-methoxyphenyl)methylamino]ethyl}acetamide (UCM793), has nanomolar binding affinity for MT_1_ and MT_2_ membrane receptors. To explore the effect of chain conformation on receptor binding, a methyl group was inserted on the methylene alpha or beta to the amide nitrogen and conformational equilibria were investigated by NMR spectroscopy and molecular dynamics simulations. Receptor affinity was conserved only for the beta-methyl derivative, which also showed significant stereoselectivity, with the (*S*) enantiomer being the eutomer. Molecular dynamics simulations, validated by NMR spectroscopy, showed that the beta-methyl group affects the conformational preferences of the ethylamide chain. Docking into the receptor crystal structure provides a rationale for the observed chiral recognition, suggesting that the (*S*)-beta-methyl group favors the conformation that better fits the receptor binding site.

## 1. Introduction

**Figure 1:**
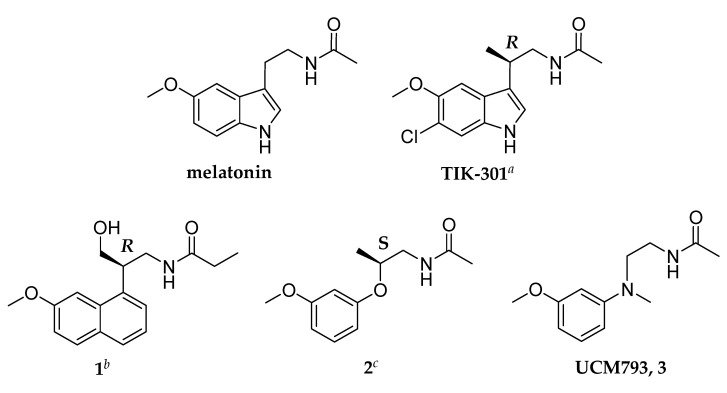
Melatonin, *N*-{2-[(3-methoxyphenyl)methylamino]ethyl}acetamide (UCM793), and melatoninergic compounds bearing modifications on the ethylamide side chain: TIK-301, (*R*)-*N*-[3-hydroxy-2-(7-methoxynaphthalen-1-yl)propyl]propanamide (1) and (*S*)-*N*-[2-(3-methoxyphenoxy)propyl]acetamide (2). *^a^ K*_i_(MT_1_) = 0.081 nM; *K*_i_(MT_2_) = 0.042 nM [7]. *^b^* p*K*_i_(MT_1_) = 8.47; p*K*_i_(MT_2_) = 9.50 [9]. *^c^* p*K*_i_(MT_1_) = 8.77; p*K*_i_(MT_2_) = 8.33 [10]. Binding affinity data for melatonin and UCM793 are reported in Table 1.

Melatonin (Figure 1) is a serotonin-derived neurohormone primarily secreted by pinealocytes, endocrine cells situated in the pineal gland, which receive signals from the suprachiasmatic nucleus to entrain circadian rhythms of vertebrates to the daily and seasonal light-dark cycle. This hormone is also produced in other tissues including the retina, the gastrointestinal tract, and different types of skin cells, and participates to a multitude of biological activities in both the central nervous system (CNS) and peripheral organs. Melatonin is involved in the regulation of the sleep-wake cycle, body temperature, and hormone secretion, and in peripheral districts it acts as a modulator of diverse physiological functions, comprising the activity of the immune and cardiovascular systems and glucose homeostasis [1]. In humans, these multiple functions are primarily mediated by the activation of MT_1_ and MT_2_ receptors, which belong to the rhodopsin-like family of G-protein-coupled receptors (GPCRs) [2]. MT_1_ and MT_2_ are coupled to inhibitory G proteins, leading to decreased intracellular levels of cAMP, even if other signal transduction mechanisms have been characterized [3]. Modulation of the melatoninergic system is considered an effective therapeutic strategy in several fields, for CNS-related pathologies, such as sleep and circadian rhythms disturbances, depression, and for other diseases including neurodegenerative diseases, type 2 diabetes, stroke, and cancer [4]. The efforts to characterize the melatoninergic system and to exploit the therapeutic potential of MT_1_ and MT_2_ receptors have led to the design and development of several ligands [5]. The melatonin bioisosteres ramelteon, agomelatine, and tasimelteon have been approved for the treatment of sleep disturbances, depression, and non-24-h sleep–wake disorder, respectively [4]. Several classes of melatoninergic ligands have been synthesized in the last decades in which modification of the relevant structural features of melatonin (i.e., the aromatic core, the methoxy group and the amide side chain) were carried out, leading to receptor ligands characterized by diverse pharmacological profiles in terms of binding affinity, receptor-subtype selectivity, and intrinsic activity [6]. Further attempts to improve ligand efficacy and metabolic stability were made by introducing substituents on the alkylamide side chain. The most advanced compound of this type is the melatonin derivative TIK-301 (LY 156735, (*R*)-β-methyl-6-chloro-melatonin, Figure 1) developed by Eli Lilly (Indianapolis, USA), which received orphan drug designation for circadian rhythm sleep disorder. A methyl group was introduced on the beta carbon of the ethylamide side chain of TIK-301, which demonstrated higher binding affinity and efficacy than melatonin [7]. Several substituents were evaluated in the beta position of the ethyl acetamide side chain of agomelatine, with methyl and fluoromethyl groups leading to an increase of receptor binding affinity [8]. Interestingly, evaluation of single enantiomers of the beta-hydroxymethyl derivative highlighted a stereoselective behavior, with the *R* enantiomer (compound **1** in Figure 1) being characterized by higher binding affinity and potency in functional assays [9]. The favorable effect on binding affinity produced by a beta-methyl group was also observed on *N*-[2-(3-methoxyphenoxy)ethyl]acetamide and its thio-analogue, which are structurally simplified derivatives of melatonin. Stereoselectivity was observed, with the *S* enantiomer being the eutomer (compound **2** in Figure 1) on both series [10]. Several ligand- and structure-based models have been applied to the investigation of structure-activity relationships (SARs) for indole-mimicking nuclei, and the *m*-methoxyanilino fragment has emerged as one of the simplest and most versatile scaffolds. As for the ethylamide chain, its active conformation had been proposed on the basis of the biological activity observed for rigid compounds [11,12], recently confirmed by the crystal structures of melatonin receptors [13,14]. On the other hand, the role of chain flexibility has not been investigated, and the reason of the chiral recognition observed for methyl derivatives is not known.

To address these points, we assessed the role of a methyl group placed on the alkylamide side chain on receptor binding affinity synthesizing and testing alpha- and beta-methyl derivatives of *N*-{2-[(3-methoxyphenyl)methylamino]ethyl}acetamide (UCM793, compound **3** in Figure 1). The parent compound belongs to a series of *N*-anilinoethylamide melatonin-receptor ligands, some of which displayed interesting sleep-inducing, antinociceptive or anxiolytic properties in appropriate animal models, without showing evident signs of toxicity [15,16]. Binding affinity, intrinsic activity, and metabolic behavior of this class of compounds can be modulated by introduction of the appropriate substituents into the aniline nucleus [17]. The small *N*-methyl group affords a nonselective MT_1_ and MT_2_ agonist with nanomolar binding affinity [18]. Docking studies into the receptor binding site were performed for the beta-methyl derivatives characterized by high binding affinity and stereoselectivity. Combination of docking pose and investigation of the conformational equilibria in solution by molecular dynamics simulations and NMR spectroscopy allowed to rationalize the stereoselective behavior shown by the two enantiomers.

## 2. Results and Discussion

### 2.1. Chemistry

**Scheme 1:**
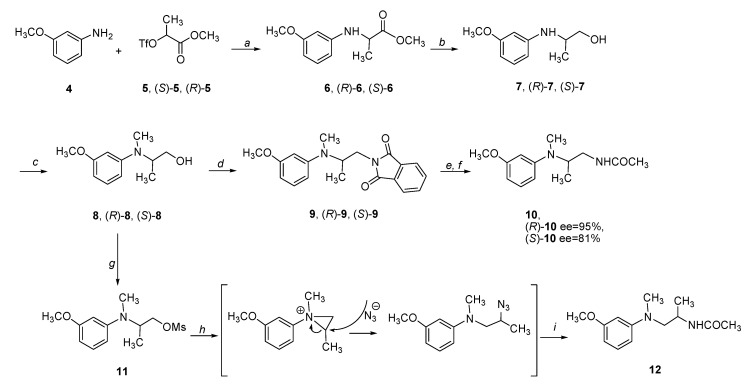
General synthesis of compounds **10** and **12**. *Reagents and conditions*: (a) dry CH_2_Cl_2_, rt, 14 h, 94–98% yield; (b) LiAlH_4_, dry THF, rt, 30 min, 88–94% yield; (c) 37% HCHO, NaBH_3_CN, CH_3_OH/AcOH, rt, 16 h, 93–99% yield; (d) DEAD, phthalimide, PPh_3_, dry THF, rt, 16 h, 51–54% yield; (e) N_2_H_4_ H_2_O, AcOH, dry CH_3_OH, 4 h, reflux; f) acetic anhydride, Et_3_N, dry THF, rt, 1 h, two steps (e, f) 90–98% yield; (g) mesyl chloride, Et_3_N, dry CH_2_Cl_2_, rt, 1 h, 72% yield; (h) NaN_3_, dry DMF, 110 °C, 3 h; (i) H_2_ (4 atm), Pd/C 10%, acetic anhydride, *i*-PrOH, rt, 6 h, two steps (h, i) 57% yield.

The target compounds 10 in their racemic and optically active form were prepared according to the synthetic route depicted in Scheme 1. *N*-alkylation of 3-methoxyaniline (4) with racemic (5) or enantiomerically pure [(*S*)-5 or (*R*)-5] methyl 2-(trifluoromethylsulfonyloxy)propanoate (in the latter case, the reaction takes place with Walden inversion at the chiral carbon atom), followed by LiAlH_4_ reduction of the obtained aminoesters 6 [19], (*R*)-6 or (*S*)-6, gave the corresponding alcohols [7, (*R*)-7 and (*S*)-7]. The *N*-methyl derivatives 8, (*R*)-8 and (*S*)-8 were prepared by reductive methylation (37% HCHO/NaBH_3_CN) of the intermediate aminoalcohols [7, (*R*)-7 and (*S*)-7], to then be condensed with phthalimide following a typical Mitsunobu procedure. Hydrazinolysis of the resulting phthalimido derivatives [9, (*R*)-9 and (*S*)-9] and *N*-acetylation of the crude amines with acetic anhydride led to the desired final products 10, (*R*)-10, and (*S*)-10. (The synthetic steps from (*R*)-6 and (*S*)-6 to (*R*)-10 and (*S*)-10, respectively, are carried out with retention of configuration of all intermediates).

The target compound **12** was synthesized through a reaction sequence involving mesylation of alcohol 8 under classic conditions (MsCl, Et_3_N, CH_2_Cl_2_, rt), subsequent treatment of mesylate **11** with sodium azide to provide an azido intermediate that was converted into the desired final compound **12** by catalytic (10% Pd/C) hydrogenation and concomitant acetylation of the crude anilinoalkylamine with acetic anhydride. The formation of compound **12** is entirely consistent with the reaction proceeding via the intermediacy of an aziridinium cation, and its subsequent regioselective ring opening (Scheme 1) [20]. The observed NH-CH_α_ correlation in the COSY spectrum is in agreement with the proposed structure of compound **12** (Supplementary Materials Figure S13).

### 2.2. Biological Activity

The newly synthesized alpha- and beta-methyl *N*-anilinoethylamides were evaluated for their binding affinity at human MT_1_ and MT_2_ receptors stably transfected in NIH3T3 cells using 2-[^125^I]iodomelatonin as radiolabelled ligand. Intrinsic activity was determined with the GTPγS test and the results are reported in Table 1. The alpha-methyl derivative **12**, tested as racemate, showed a significant decrease of binding affinity at both receptors compared to the unsubstituted compound **3**. This result, similar to what had been observed for phenoxy-ethyl-amide derivatives [10], discouraged from further investigations in this direction. On the other hand, the racemic beta-methyl derivative **10** showed binding affinity and agonist activity at both receptors comparable to those recorded for the unsubstituted compound **3**. To further explore the potency and selectivity of compound **10**, each enantiomer was independently prepared and separately tested. Even if partial racemization allowed us to test the (*S*) and (*R*) enantiomer with enantiomeric excess of 95 and 81%, respectively, selectivity in chiral recognition was evidenced, with about tenfold higher binding affinity measured for the (*S*) enantiomer at both receptor subtypes.

### 2.3. Docking of Beta-Methyl Derivatives (S)-10 and (R)-10 into MT_2_ Receptor Crystal Structure

**Figure 2:**
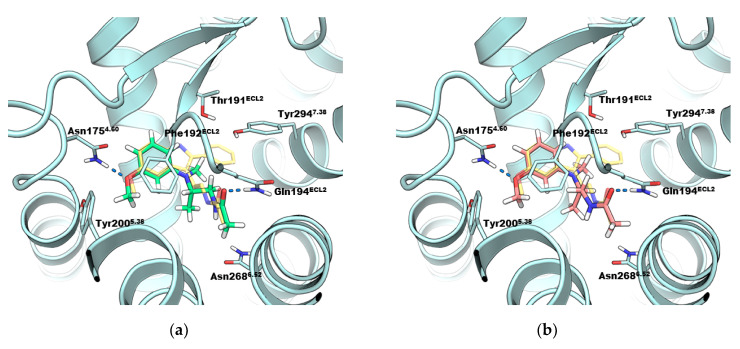
Proposed binding mode for compounds (*S*)-**10** (**a**), green sticks and (*R*)-**10** (**b**), orange sticks into the MT_2_ receptor crystal structure (PDB: 6ME6), observed from the extracellular side of the receptor. Hydrogen bonds are shown as blue dashed lines. Co-crystalized 2-phenylmelatonin is shown with shaded yellow carbons. Ballesteros-Weinstein residue numbering [22] is adopted in superscripts.

Compounds (*S*)-**10** and (*R*)-**10** were docked into the crystal structure of the MT_2_ receptor [14], given the higher binding affinity shown for this subtype over MT_1_. The best poses obtained for the two enantiomers, represented in Figure 2, had similar GScore values. They undertake the same polar interactions as melatonin with receptor residues, i.e., hydrogen bonds established between the methoxy oxygen and the side chain of Asn175^4.60^ in transmembrane helix (TM) 4, and between the amide carbonyl and Gln194 belonging to the second extracellular loop (ECL2). Moreover, the phenyl ring and the side chain of Phe192 on ECL2 form a π-stacking interaction.

**Figure 3:**
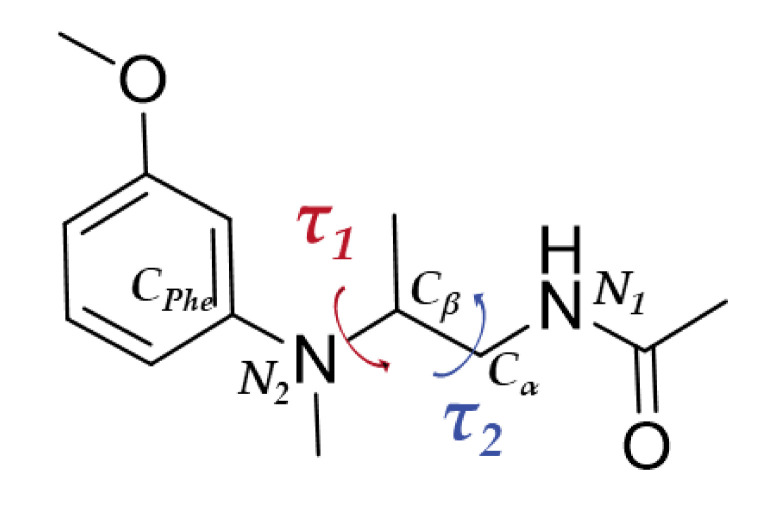
Representation of τ_1_ (C_Phe_–N_2_–C_β_–C_α_) and τ_2_ (N_2_–C_β_–C_α_–N_1_) dihedral angles.

Both enantiomers place the methyl group within the same hydrophobic region, which is open toward the intracellular portion of the receptor and surrounded by hydrophobic amino acids (Trp264^6.48^, Val204^5.42^, Val124^3.36^, and Ile125^3.37^, not shown in Figure 2). To place the methyl group in the same pocket while the methoxyanilino scaffold and the acetamide take the same interactions, the two enantiomers adopt different conformations. In particular, referring to the dihedral angles τ_1_ and τ_2_ as defined in Figure 3, the eutomer (*S*)-**10** has τ_1_ = −135° and τ_2_ = 177°, while the distomer (*R*)-**10** has τ_1_ = −52° and τ_2_ = −174° after energy minimization of the ligand-receptor complex.

**Figure 4:**
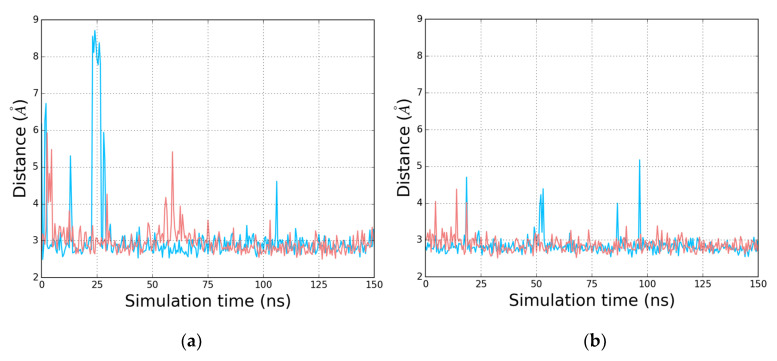
Distance plots for polar interactions between residues from the MT_2_ receptor and (*S*)-**10** (**a**) or (*R*)-**10** (**b**) during 150 ns of molecular dynamics (MD) simulation of the ligand-receptor complexes. The distance between the methoxy oxygen of the ligand and the amide nitrogen of Asn175^4.60^ is shown in orange; the distance between the amide carbonyl oxygen of the ligand and the amide nitrogen of Gln194^ECL2^ is shown in cyan.

**Figure 5:**
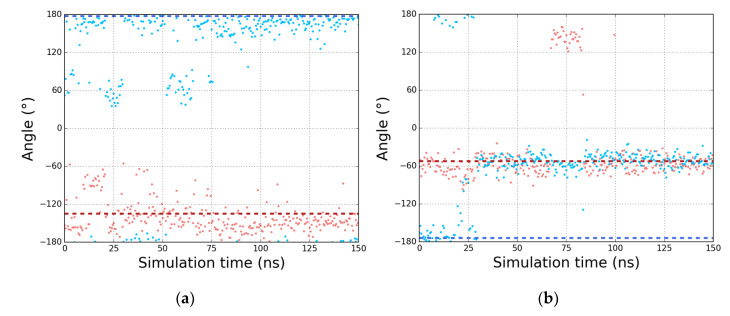
Values of dihedral angles τ_1_ (orange dots) and τ_2_ (cyan dots) (defined in Figure 3) monitored during 150 ns MD simulation for (*S*)-**10** (**a**) and (*R*)-**10** (**b**) in complex with the MT_2_ receptor. Dashed lines represent the dihedral values τ_1_ (red) and τ_2_ (blue) assumed in the minimized docked poses.

The two energy-minimized complexes were submitted to 150 ns of molecular dynamics (MD) simulation to test their dynamic stability. The main hydrogen bonds between ligand and protein (i.e., *i*. between the methoxy oxygen of the ligand and the amide nitrogen of Asn175^4.60^; *ii*. between the amide carbonyl oxygen of the ligand and the amide nitrogen of Gln194^ECL2^) were generally maintained throughout the simulation (Figure 4). For both enantiomers, the dihedral angle τ_1_ fluctuated around the docking values, and τ_2_ assumed two different values, both allowing the hydrogen bond between the amide group and Gln194^ECL2^ (Figure 5).

The docking results did not explain the moderate, yet significant, chiral recognition observed for the beta-methyl derivatives at the MT_2_ receptor. The stability of the two complexes was similar and the docking scores were very similar as well (GScore = −8.602 for (*S*)-**10** and −8.413 kcal·mol^−1^ for (*R*)-**10**). To estimate the binding free energies of the two enantiomers, we performed generalized Born and surface area continuum solvation (MM-GBSA) calculations following a protocol previously applied to drug-protein interactions [23]. The estimated binding energies were very similar for the two enantiomers (−59.02 for (*S*)-**10** and −57.76 kcal·mol^−1^ for (*R*)-**10**) confirming that chiral recognition does not derive from a better accommodation of the chiral center within the receptor.

Thus, we hypothesized that the beta-methyl group could alter the conformational equilibria of the unsubstituted precursor and induce a conformational selection, which could favor certain conformations. According to this hypothesis, the bioactive conformation of the eutomer (*S*)-**10** should be more abundant than the corresponding one of the distomer (*R*)-**10**.

### 2.4. Conformational Equilibria of Beta-Methyl Derivatives in Solution: MD Simulations and NMR Spectroscopy

**Figure 6:**
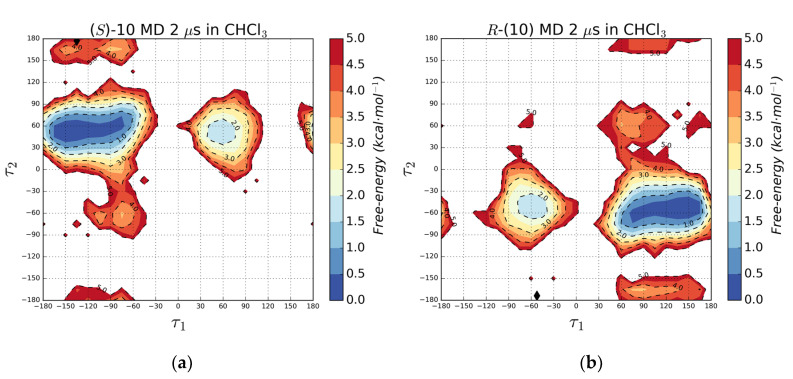
Free energy surfaces for (*S*)-**10** (**a**) and (*R*)-**10** (**b**) after 2 µs of molecular dynamics simulation in chloroform. Dihedral values assumed in the minimized docking poses are shown as black diamonds.

To assess the available conformational space of (*R*)-**10** and (*S*)-**10** in solution, a 2-µs-long MD simulation in a box of explicit chloroform was performed for each enantiomer. The free energy minimum for (*S*)-**10** corresponds to τ_1_~−150° and τ_2_~60°, and the same result, with specular angle values, was obtained for (*R*)-**10**, which showed the most populated conformation at τ_1_~150° and τ_2_~−60° (Figure 6).

**Figure 7:**
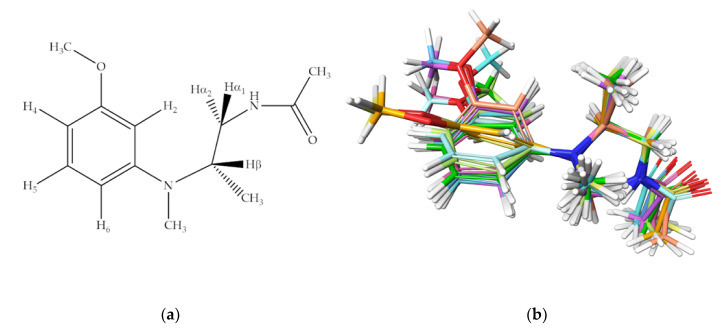
Structure of (*S*)-**10** with proton labels (**a**,**b**) superposition of 10 frames from MD simulation of (*S*)-**10** in chloroform taken from the minimum-energy cluster represented in Figure 6 (τ_1_~−150° and τ_2_~60°).

The global minimum identified by MD simulations in chloroform is consistent with results from NMR spectroscopy investigation. The spectrum obtained for the racemate in deuterated chloroform at 600 MHz allowed the assignment of all peaks to the protons and analysis of the coupling constants and NOE signals revealed reduced conformational freedom of the ethylamide side chain (see Supplementary Materials for NMR spectra). In fact, the signal of the beta-hydrogen has two coupling constants (J_Hβ,Hα1_ = 4.9 Hz and J_Hβ,Hα2_ = 10.7 Hz; see Supplementary Materials Figure S8 and Figure 7 for proton labels), which indicates that τ_2_ dihedral angle assumes a preferred value with the beta proton (Hβ) in gauche arrangement with one alpha hydrogen (Hα_1_ in Figure 7) and antiperiplanar to the other (Hα_2_). A strong NOE signal is visible between Hβ and Hα_1_ (gauche), while there is no such evidence for Hβ and Hα_2_ (antiperiplanar). Moreover, an intense NOE cross-peak indicates that the two methyl groups NCH_3_ e CHCH_3_ lie in close proximity, while no contacts were recorded between NCH_3_ and Hβ. Furthermore, Hβ showed NOE contacts with the aromatic protons H2 and H6, while no contact was observed between the beta-methyl group and the aromatic ring (Supplementary Materials Figure S10). Overall, these signals support an arrangement of the beta-hydrogen (Hβ) in the plane of the aromatic ring, in opposite direction to NCH_3_, while the beta-methyl group is placed roughly perpendicular to the aromatic ring, close to the NCH_3_ group. These data identify a preferred conformation having τ_1_~−150° and τ_2_~60° (depicted in Figure 7b) for compound (*S*)-**10**, corresponding to the global free energy minimum obtained from the MD simulation. Simulations in a water box gave similar results (Figure S20 in Supplementary Materials), confirming that the beta-methyl group induces an asymmetric probability distribution for the chain conformations around τ_1_, placing the amide moiety over or beneath the plane identified by the anilino scaffold.

Comparing the conformations of the two enantiomers in complex with the MT_2_ receptor, (*S*)-**10** interacts with the binding site residues assuming the value of τ_1_ dihedral angle of ~−150°, which is also preferred in solution (Figure 6a), while the τ_1_ value of the receptor-docked conformation of (*R*)-**10** (~−60°) is significantly disfavored (Figure 6b). In both the ligand-receptor complexes, τ_2_ has an anti-arrangement, differing from the gauche (~60° and ~−60° for (*S*)-**10** and (*R*)-**10**, respectively), which is the most abundant one in solution. While the conformation with τ_2_~180° is populated with low abundance in the MD simulation in chloroform, interaction with the receptor is presumed to enforce the anti-arrangement to allow a hydrogen bond between the amide carbonyl and Gln194^ECL2^. Moreover, the micro-environment at membrane-receptor boundaries could affect the conformational arrangement of the amide fragment around τ_2._ In this regard, while the MD simulation in a chloroform box (a lipophilic solvent that allowed direct comparison with NMR data) showed for (*S*)-**10** a free energy for the docking conformation (τ_1_~−150°, τ_2_~180°) higher than the global minimum by approximately 3 kcal·mol^−1^, a similar simulation in a water box (Figure S20 in Supplementary Materials) showed even higher free energy. Conversely, a simulation in 1-palmitoyl-2-oleoyl-*sn*-phosphatidylcholine (POPC) gave a slightly lower free energy difference between the two basins. While speculative, this result is consistent with molecular dynamics [24] and metadynamics [25] simulations performed by Martì and colleagues, who suggest that the ligand could be concentrated via the polar heads interface at the membrane-receptor boundary, and with dissociation rates measured on different receptor mutants, which suggest a preferred ligand entry into the binding site through the membrane opening between TM4 and TM5 [14].

Importantly, while the situation for τ_2_ is similar for the two enantiomers (the gauche arrangement is preferred in solution, the anti-one within the receptor), in the *S* enantiomer the introduction of the methyl group in beta position of the ethylamide side chain induces a conformational enrichment of the conformation with the value of τ_1_ observed in the docking pose. This can explain the moderate chiral recognition observed for the beta-methyl derivative. Another important point is that the high receptor affinity shown by the two enantiomers can be explained assuming that the beta-methyl group is accommodated in an additional lipophilic pocket, just under the ligand binding site, which is detectable within the crystal structures now available and can explain the good binding affinities previously reported for melatoninergic ligands such as TIK-301 and compounds **1** and **2**. This additional pocket could be exploited for the design of novel melatoninergic agents with different therapeutic applications.

## 3. Materials and Methods

### 3.1. Chemistry

#### General Procedures

Column chromatography purifications were performed under “flash” conditions using Merck 230−400 mesh silica gel. Analytical thin-layer chromatography (TLC) was carried out on Merck silica gel 60 F_254_ plates. ^1^H NMR and ^13^C NMR spectra were recorded on a Bruker AVANCE 200, 400 or on a Varian INOVA 600 MHz spectrometer using CDCl_3_ as solvent. Chemical shifts (δ scale) are reported in parts per million (ppm) relative to the central peak of the solvent. Coupling constants (*J*) are given in hertz (Hz). ESI MS spectra were taken on a Waters Micromass ZQ instrument; only molecular ions (M + 1)^+^ are given. Elemental analyses were performed on a Perkin-Elmer 2400 elemental analyzer, and the data for C, H, and N were within ±0.4% of the theoretical values. Optical rotation analysis was performed using a Perkin-Elmer 241 polarimeter using a sodium lamp (λ 589 nm, D-line), α values were determined at 20 °C and are reported in 10^−1^ deg cm^2^ g^−1^; concentration (*c*) is in g per 100 mL. Enantiomeric purity was determined by HPLC and performed on a AD-H-Chiralpak or OD-H-Chiralcel column (φ = 0.46 cm, l = 25 cm, 5 μm) on the following apparatus: Shimadzu LC-10AT (liquid chromatograph), Shimadzu SPD-10A (UV detector), Shimadzu C-R6A Chromatopac (integrator).

*(R*,*S*)-methyl 2-(trifluoromethylsulfonyloxy)propanoate (**5**) was prepared according to a previously reported procedure for the corresponding (2*R*)-enantiomer [26]. Spectroscopic data were in agreement with those reported for the (2*R*)-enantiomer.

*(R,S)-Methyl 2-(3-methoxyphenylamino)propanoate* (**6**), a solution of **5** (1.18 g, 5 mmol) in dry CH_2_Cl_2_ (20 mL) was added to an ice-cooled solution of 3-methoxyaniline (1.23 g, 10 mmol) in dry CH_2_Cl_2_ (20 mL) under nitrogen atmosphere, and the resulting mixture was stirred at room temperature for 14 h. The reaction mixture was filtered and the filtrate partitioned between water and CH_2_Cl_2_. The combined organic phases were washed with brine, dried (Na_2_SO_4_), and concentrated by distillation under reduced pressure to give a crude residue, which was purified by flash chromatography (silica gel, cyclohexane/EtOAc 8:2 as eluent). Oil; 98% yield [19]. ^1^H NMR (200 MHz, CDCl_3_) δ 1.48 (d, 3H, *J* = 7.0 Hz, CHCH_3_), 3.75 (s, 3H, OCH_3_), 3.78 (s, 3H, OCH_3_), 4.15 (q, 1H, *J* = 6.9 Hz, CHCH_3_), 6.17–6.35 (m, 3H, Ar), 7.10 (dd, 1H, *J_1_* = *J_2_* = 8.0 Hz, Ar). ^13^C NMR (100 MHz, CDCl_3_) δ 175.0, 160.8, 147.9, 130.1, 106.3, 103.5, 99.4, 55.1, 52.3, 51.9, 18.9. ESI MS (*m*/*z*) 210 (M + H)^+^.

*(R)-Methyl 2-(3-methoxyphenylamino)propanoate* [(*R*)-**6**], prepared following the above described procedure for the synthesis of **6**, starting from (*S*)-**5 [27]**. Oil; 95% yield. ^1^H NMR and ESI MS data were in agreement with those reported for compound **6**. [α]^20^_D_ = −48.06° (*c* 0.206 in CHCl_3_); 95% ee, determined on AD-H column: *n*-hexane/*i*-PrOH 98:2 as eluent, λ 290 nm, flow rate of 1.0 mL/min and t_R_ 22.59 min.

*(S)-Methyl 2-(3-methoxyphenylamino)propanoate* [(*S*)-**6**], prepared following the above described procedure for the synthesis of **6**, starting from (***R***)-**5 [26]**. Oil; 94% yield. ^1^H NMR and ESI MS data were in agreement with those reported for compound **6**. [α]^20^_D_ = +48.40° (*c* 0.215 in CHCl_3_); 96% ee, determined on AD-H column: *n*-hexane/*i*-PrOH 98:2 as eluent, λ 290 nm, flow rate of 1.0 mL/min and t_R_ 30.69 min.

*(R,S)-2-(3-Methoxyphenylamino)propan-1-ol* (**7**), a solution of **6** (420 mg, 2 mmol) in dry THF (10 mL) was added dropwise to a stirred and ice-cooled suspension of LiAlH_4_ (240 mg, 2 mmol) in dry THF (10 mL) under nitrogen atmosphere, and the resulting mixture was stirred at room temperature for 30 min. EtOAc and water were carefully added at 0 °C, and the resulting mixture was filtered through a Celite pad. The filtrate was extracted (3×) with EtOAc, the combined organic phases were washed with brine, dried (Na_2_SO_4_), and concentrated by distillation under reduced pressure to yield a crude residue, which was purified by flash chromatography (silica gel, cyclohexane/EtOAc 6:4 as eluent). Oil; 92% yield. ^1^H NMR (400 MHz, CDCl_3_) δ 1.20 (d, 3H, *J* = 5.5 Hz, CHCH_3_), 3.51 (dd, 1H, *J* = 6.0, 10.5 Hz, CH_2_OH), 3.59–3.66 (m, 1H, CHCH_3_), 3.72 (dd, 1H, *J* = 4.5, 10.5 Hz, CH_2_OH), 3.78 (s, 3H, OCH_3_), 6.23 (dd, 1H, *J_1_* = *J_2_* = 2.5 Hz, Ar), 6.28 (dd, 1H, *J* = 2.5, 8.0 Hz, Ar), 6.31 (dd, 1H, *J* = 2.5, 8.0 Hz, Ar), 7.09 (dd, 1H, *J_1_* = *J_2_* = 8.0 Hz, Ar). ^13^C NMR (100 MHz, CDCl_3_) δ 160.9, 148.8, 130.1, 106.8, 103.0, 99.8, 66.1, 55.1, 50.6, 17.5. ESI MS (*m*/*z*) 182 (M + H)^+^.

*(R)-2-(3-Methoxyphenylamino)propan-1-ol* [(*R*)-**7**], prepared following the above described procedure for the synthesis of **7**, starting from (*R*)-**6**. Oil; 94% yield. ^1^H NMR and ESI MS data were in agreement with those reported for compound **7**. [α]^20^_D_ = −29.95° (*c* 0.163 in CHCl_3_); 95% ee, determined on OD-H column: *n*-hexane/*i*-PrOH 8:2 as eluent, λ 290 nm, flow rate of 0.7 mL/min and t_R_ 16.50 min.

*(S)-2-(3-Methoxyphenylamino)propan-1-ol* [(*S*)-**7**], prepared following the above described procedure for the synthesis of **7**, starting from (*S*)-**6**. Oil; 88% yield. ^1^H NMR and ESI MS data were in agreement with those reported for compound **7**. [α]^20^_D_ = +30.26° (*c* 0.154 in CHCl_3_); 92% ee, determined on OD-H column: *n*-hexane/*i*-PrOH 8:2 as eluent, λ 290 nm, flow rate of 0.7 mL/min and t_R_ 15.00 min.

*(R,S)-2-[(3-Methoxyphenyl)-(methyl)amino]propan-1-ol* (**8**), a 37% aqueous formaldehyde solution (0.9 mL, 11.1 mmol) was added to a solution of **7** (273 mg, 1.5 mmol), sodium cyanoborohydride (114 mg, 1.8 mmol) and glacial acetic acid (0.42 mL, 7.2 mmol) in CH_3_OH (9 mL), and the resulting mixture was stirred at room temperature for 16 h. Methanol was removed by distillation under reduced pressure, the mixture was basified with 30% NaOH end extracted (3×) with CH_2_Cl_2_. The combined organic phases were washed with brine, dried (Na_2_SO_4_) and concentrated by distillation under reduced pressure to give a crude residue, which was purified by flash chromatography (silica gel, cyclohexane/EtOAc 8:2 as eluent). Oil; 95% yield. ^1^H NMR (200 MHz, CDCl_3_) δ 1.05 (d, 3H; *J* = 6.5 Hz, CHCH_3_), 2.10 (brs, 1H, OH), 2.73 (s, 3H, NCH_3_), 3.57–3.70 (m, 2H, CH_2_OH), 3.81 (s, 3H, OCH_3_), 3.97–4.14 (m, 1H, CHCH_3_ ), 6.39 (dd, 1H, *J* =2.5, 8.0 Hz, Ar), 6.48 (dd, 1H, *J_1_* = *J_2_* = 2.5 Hz, Ar), 6.57 (dd, 1H, *J* =2.5, 8.0 Hz, Ar), 7.18 (dd, 1H, *J_1_* = *J_2_* = 8.0 Hz, Ar). ESI MS (*m*/*z*) 196 (M + H)^+^.

*(R)-2-[(3-Methoxyphenyl)-(methyl)amino]propan-1-ol* [(*R*)-**8**], prepared following the above described procedure for the synthesis of **8**, starting from (*R*)-**7**. Oil; 99% yield. ^1^H NMR and ESI MS data were in agreement with those reported for compound **8**. [α]^20^_D_ = +66.18° (*c* 0.319 in CHCl_3_).

*(S)-2-[(3-Methoxyphenyl)-(methyl)amino]propan-1-ol* [(*S*)-**8**], prepared following the above described procedure for the synthesis of **8**, starting from (*S*)-**7**. Oil; 93% yield. ^1^H NMR and ESI MS data were in agreement with those reported for compound **8**. [α]^20^_D_ = −66.70° (*c* 0.411 in CHCl_3_).

(*R,S*)-2-{2-[(3-Methoxyphenyl)-(methyl)amino]propyl}isoindoline-1,2-dione (**9**), a solution of diethyl azodicarboxylate (DEAD, 230 mg, 1.3 mmol) in dry THF (6 mL) was added to a stirred ice-cooled solution of **8** (170 mg, 0.9 mmol), phthalimide (193 mg, 1.3 mmol), and PPh_3_ (320 mg, 1.3 mmol) in dry THF (3 mL) under nitrogen atmosphere. The resulting mixture was stirred at room temperature for 16 h, and then was concentrated by distillation under reduced pressure to give a crude residue that was purified by flash chromatography (silica gel, cyclohexane/EtOAc 8:2 as eluent). Yellow oil; 54% yield. ^1^H NMR (200 MHz, CDCl_3_) δ 1.24 (d, 3H *J* = 6.5 Hz, CHCH_3_), 2.78 (s, 3H, NCH_3_), 3.61 (dd, 1H *J* = 6.5, 14.0 Hz, CH_2_-Pht), 3.72 (s, 3H, OCH_3_), 3.94 (dd, 1H *J* = 9.5,14.0 Hz, CH_2_-Pht), 4.45–4.63 (m, 1H, CHCH_3_), 6.09 (dd, 1H, *J* = 2.0, 8.0 Hz, Ar), 6.30–6.39 (m, 2H, Ar), 6.96 (dd, 1H *J_1_* = *J_2_* = 8.0, Ar), 7.63–7.78 (m, 4H, Ar-Pht). ^13^C NMR (100 MHz, CDCl_3_) δ 168.4, 160.5, 151.7, 133.8, 131.9, 129.6, 123.1, 106.6, 102.3, 99.8, 54.9, 52.0, 40.3, 30.1, 15.4. ESI MS (*m*/*z*) 325 (M + H)^+^.

*(R)-2-{2-[(3-Methoxyphenyl)-(methyl)amino]propyl}isoindoline-1,2-dione* [(*R*)-**9**], prepared following the above described procedure for the synthesis of **9**, starting from (*R*)-**8**. Oil; 51% yield. ^1^H NMR and ESI MS data were in agreement with those reported for compound **9**. [α]^20^_D_ = −74.13° (*c* 0.174 in CHCl_3_); 94% ee, determined on OD-H column: *n*-hexane/*i*-PrOH 8:2 as eluent, λ 290 nm, flow rate of 0.7 mL/min and t_R_ 16.10 min.

*(S)-2-{2-[(3-Methoxyphenyl)-(methyl)amino]propyl}isoindoline-1,2-dione* [(*S*)-**9**], prepared following the above-described procedure for the synthesis of **9**, starting from (*S*)-**8**. Oil; 52% yield. ^1^H NMR and ESI MS data were in agreement with those reported for compound **9**. [α]^20^_D_ = +64.4° (*c* 0.180 in CHCl_3_); 85% ee, determined on OD-H column: *n*-hexane/*i*-PrOH 8:2 as eluent, λ 290 nm, flow rate of 0.7 mL/min, and t_R_ 12.80 min.

*(R,S)-N-{2-[(3-Methoxyphenyl)-(methyl)amino]propyl}acetamide* (**10**), glacial acetic acid (0.05 mL, 0.9 mmol) and 65% hydrazine hydrate (0.13 mL, 1.8 mmol) were added to a solution of **9** (150 mg, 0.45 mmol) in dry CH_3_OH (5 mL) and the resulting mixture was stirred under reflux for 4 h. After cooling to room temperature, the solid was filtered off and the filtrate was concentrated by distillation under reduced pressure to yield a residue, which was taken up with EtOAc and extracted twice with 2N HCl. The combined aqueous phases were basified with a saturated aqueous NaHCO_3_ solution, and extracted (3×) with EtOAc. The combined organic phases were washed with brine, dried (Na_2_SO_4_), and concentrated by distillation under reduced pressure to give the crude primary amine, which was used without any further purification. Oil. ESI MS (*m*/*z*) 195 (M + H)^+^.

Acetic anhydride (0.05 mL, 0.5 mmol) was added to a solution of the above crude amine and Et_3_N (0.09 mL, 0.66 mmol) in dry THF (3 mL), and the resulting mixture was stirred 1 h at room temperature. After removing the solvent by distillation under reduced pressure, the residue was purified by flash chromatography (silica gel, cyclohexane/EtOAc 3:7 to 100% EtOAc as eluent). Oil; 93% yield. ^1^H NMR (600 MHz, CDCl_3_) δ 1.09 (d, 3H, *J* = 6.7 Hz, CHCH_3_), 1.89 (s, 3H, COCH_3_), 2.68 (s, 3H, NCH_3_), 3.13 (ddd, *J* = 13.5, 10.7, 2.9 Hz, Hα_2_, see Figure 7 for proton labels), 3.55 (ddd, *J* = 13.8, 7.1, 4.9 Hz, Hα_1_), 3.77 (s, 3H, OCH_3_), 4.04 (dqd, 1H, *J* = 11.5, 6.4, 5.1 Hz, Hβ), 5.63 (brs, 1H, NH), 6.32 (d, 1H *J* = 8.0 Hz, H4), 6.37 (s, 1H, H2), 6.45 (d, 1H *J* = 8.5 Hz, H6) 7.13 (dd, 1H *J* = 8.0 and *J* = 8.5 Hz, H5). ^13^C NMR (100 MHz, CDCl_3_) δ 170.2, 160.8, 152.1, 129.9, 107.1, 102.4, 100.7, 55.2, 53.7, 42.3, 29.9, 23.2, 14.4. ESI MS (*m*/*z*) 237 (M + H)^+^. Anal. Calcd. for C_13_H_20_N_2_O_2_ (236.31): C, 66.07; H, 8.53; N, 11.85. Found: C, 66.03; H, 8.53; N, 11.81%.

(*R*)-*N*-{2-[(3-Methoxyphenyl)-(methyl)amino]propyl}acetamide [(*R*)-**10**], prepared following the above-described procedure for the synthesis of **10**, starting from (*R*)-**9**. Oil; 90% yield. ^1^H NMR and ESI MS data were in agreement with those reported for compound **10**. Anal. Calcd. for C_13_H_20_N_2_O_2_ (236.31): C, 66.07; H, 8.53; N, 11.85. Found: C, 65.95; H, 8.54; N, 11.72%. [α]^20^_D_ = +18.4° (*c* 0.100 in CHCl_3_); 95% ee, determined on OD-H column: *n*-hexane/*i*-PrOH 8:2 as eluent, λ 298 nm, flow rate of 0.7 mL/min, and t_R_ 19.20 min.

*(S)-N-{2-[(3-Methoxyphenyl)-(methyl)amino]propyl}acetamide* [(*S*)-**10**], prepared following the above-described procedure for the synthesis of **10**, starting from (*S*)-**9**. Oil; 98% yield. ^1^H NMR and ESI MS data were in agreement with those reported for compound **10**. Anal. Calcd. for C_13_H_20_N_2_O_2_ (236.31): C, 66.07; H, 8.53; N, 11.85. Found: C, 66.01; H, 8.53; N, 11.79%. [α]^20^_D_ = −13.3° (*c* 0.050 in CHCl_3_). 81% ee, determined on OD-H column: *n*-hexane/*i*-PrOH 8:2 as eluent, λ 298 nm, flow rate of 0.7 mL/min, and t_R_ 22.84 min.

*(R,S)-2-[(3-Methoxyphenyl)-(methyl)amino]methanesulfonate* (**11**), mesyl chloride (120 mg, 1.11 mmol) was added to a solution of **8** (180 mg, 0.9 mmol) and Et_3_N (0.14 mL, 0.99 mmol) in dry CH_2_Cl_2_ (2 mL) under nitrogen atmosphere, and the resulting mixture was stirred 1 h at room temperature. Water was added and the aqueous layer was extracted three time with CH_2_Cl_2_. The combined organic extracts were washed with brine, dried (Na_2_SO_4_), and concentrated by distillation under reduced pressure to give a crude residue that was purified by flash chromatography (silica gel, cyclohexane/EtOAc 8:2 as eluent). Oil; 72% yield. ^1^H NMR (200 MHz, CDCl_3_) δ 1.25 (d, 3H, *J* = 6.5 Hz, CHCH_3_), 2.79 (s, 3H, NCH_3_), 2.92 (s, 3H, SCH_3_), 3.81 (s, 3H, OCH_3_), 4.08–4.39 (m, 3H, CHCH_2_OMs), 6.30 (dd, 1H, *J* =2.5, 8.0 Hz, Ar), 6.35–6.41 (m, 1H, Ar), 6.47 (dd, 1H, *J* =2.5, 8.5 Hz, Ar), 7.17 (dd, 1H, *J* = 8.0, 8.5 Hz, Ar), ESI MS (*m/z*) 274 (M + H)^+^.

*(R,S)-N-{1-[(3-Methoxyphenyl)-(methyl)amino]propan-2-yl}acetamide* (**12**), NaN_3_ (88 mg, 1.34 mmol) was added to a solution of **11** (180 mg, 0.66 mmol) in dry DMF (3 mL), and the resulting mixture was stirred at 110 °C for 3 h under nitrogen atmosphere. Water was added and the aqueous layer was extracted three time with EtOAc. The combined organic phases were washed with brine, dried (Na_2_SO_4_), and concentrated by distillation under reduced pressure to give the corresponding crude azide, which was used for the next step without any further purification. ESI MS (*m*/*z*) 221 (M + H)^+^.

A solution of the above crude azide in *i*-PrOH (10.5 mL) and acetic anhydride (0.6 mL, 5.9 mmol) was hydrogenated over Pd/C 10% (30 mg) at 4 atm of H_2_ for 6 h at room temperature. The catalyst was filtered on Celite, the filtrate was concentrated by distillation under reduced pressure to give a crude residue that was purified by flash chromatography (silica gel, cyclohexane/EtOAc 3:7 as eluent). Oil; 57% yield. ^1^H NMR (200 MHz, CDCl_3_) δ1.19 (d, 3H *J* = 6.5 Hz, CHCH_3_), 1.88 (s, 3H, COCH_3_), 2.96 (s, 3H, NCH_3_), 3.11 (dd, 1H *J* = 7.5, 14.5 Hz, CH_2_N), 3.57 (dd, 1H *J* = 6.0, 14.5 Hz, CH_2_N), 3.81 (s, 3H, OCH_3_), 4.25–4.32 (m, 1H, CHCH_3_), 5.37 (brs, 1H, NH), 6.29 (dd, 1H *J* = 2.0, 8.0 Hz, Ar), 6.37–6.43 (m, 2H, Ar), 7.14 (dd, 1H, *J_1_* = *J_2_* = 8.0 Hz, Ar). ^13^C NMR (100 MHz, CDCl_3_) δ 169.7, 160.8, 151.0, 129.9, 105.3, 101.6, 98.7, 57.5, 55.2, 44.6, 39.3, 23.4, 18.8. ESI MS (*m*/*z*) 237 (M + H)^+^. Anal. Calcd. for C_13_H_20_N_2_O_2_ (236.31): C, 66.07; H, 8.53; N, 11.85. Found: C, 65.90; H, 8.54; N, 11.68%.

### 3.2. Pharmacology

#### 3.2.1. Reagents

The radioligands 2-[^125^I]iodomelatonin (specific activity, 2000 Ci/mmol) and [^35^S]GTPγS ([^35^S]guanosine 5′-*O*-(3-thiotriphosphate); specific activity, 1000 Ci/mmol) were purchased from Perkin-Elmer (Milano, Italy).

#### 3.2.2. Melatonin Receptor Binding and Intrinsic Activity Evaluation

Binding affinities were determined using 2- [^125^I]iodomelatonin as the labeled ligand in competition experiments on cloned human MT_1_ and MT_2_ receptors expressed in NIH3T3 rat fibroblast cells. The characterization of NIH3T3-MT_1_ and -MT_2_ cells had been already described in detail [28,29]. Membranes were incubated for 90 min at 37 °C in binding buffer (Tris-HCl, 50 mM, pH 7.4). The final membrane concentration was 5−10 μg of protein per tube. The membrane protein level was determined in accordance with a previously reported method [30]. 2-[^125^I]Iodomelatonin (100 pM) and different concentrations of melatonin (10^−10^−10^−6^ M) or of the new compounds were incubated with the receptor preparation for 90 min at 37 °C. Nonspecific binding was assessed with 10 μM melatonin; IC_50_ values were determined by nonlinear fitting strategies with the program PRISM (GraphPad Software Inc., San Diego, CA, USA). The pK*i* values were calculated from the IC_50_ values in accordance with the Cheng−Prusoff equation [21]. The p*Ki* values are the mean of at least three independent determinations performed in duplicate.

To define the functional activity of the new compounds at MT_1_ and MT_2_ receptor subtypes, [^35^S]GTPγS binding assays in NIH3T3 cells expressing human-cloned MT_1_ or MT_2_ receptors were performed. The amount of bound [^35^S]GTPγS is proportional to the level of the analogue-induced G-protein activation and is related to the intrinsic activity of the compound under study. The detailed description and validation of this method were reported elsewhere [28,29]. Membranes (15−25 μg of protein, final incubation volume 100 μL) were incubated at 30 °C for 30 min in the presence and in the absence of melatonin analogues in an assay buffer consisting of [^35^S]GTPγS (0.3−0.5 nM), GDP (50 μM), NaCl (100 mM), and MgCl_2_ (3 mM). Nonspecific binding was defined using [^35^S]GTPγS (10 μM). In cell lines expressing human MT_1_ or MT_2_ receptors, melatonin produced a concentration-dependent stimulation of basal [^35^S]GTPγS binding with a maximal stimulation, above basal levels, of 370% and 250% in MT_1_ and MT_2_ receptors, respectively. Basal stimulation is the amount of [^35^S]GTPγS specifically bound in the absence of compounds, and it was taken as 100%. The maximal G-protein activation was measured in each experiment by using melatonin (100 nM). Compounds were added at three different concentrations (one concentration was equivalent to 100 nM melatonin, a second one 10 times smaller, and a third one 10 times larger), and the percent stimulation above basal was determined. The equivalent concentration was estimated on the basis of the ratio of the affinity of the test compound to that of melatonin. It was assumed that at the equivalent concentration the test compound occupies the same number of receptors as 100 nM melatonin. All of the measurements were performed in triplicate. The relative intrinsic activity (IAr) values were obtained by dividing the maximum ligand-induced stimulation of [^35^S]GTPγS binding by that of melatonin as measured in the same experiment. By convention, the natural ligand melatonin has an efficacy (Emax) of 100%. Full agonists stimulate [^35^S]GTPγS binding with a maximum efficacy, close to that of melatonin itself. If Emax is between 30 and 70% of that for melatonin (0.3 < IAr < 0.7), the compound is considered a partial agonist, whereas if Emax is lower than 30% (IAr < 0.3), the compound is considered an antagonist [31].

### 3.3. Molecular Modeling

#### 3.3.1. Protein Preparation

The crystal structure of the MT_2_ receptor in complex with 2-phenylmelatonin (PDB: 6ME6) was used for simulations [14]. The missing intracellular loop 3 (R232-L240) and the unsolved side chains were added with Modeller 9.21 [32]. One hundred models were generated leaving residues R231 and C241, adjacent to the reconstructed loop, flexible to allow proper geometries of the construct. Models were ranked according to the molecular probability density function (molpdf) [33] implemented in Modeller. Residues encompassing the *N*-terminal sequence of the MT_2_ receptor, linked to the thermostabilized apocytochrome b562RIL, were removed, leaving P36 as the first residue. Mutated amino acids in the crystallized protein were reverted to wild-type residues, comprising Trp246^6.48^ in the ligand binding site. The structure with the lowest molpdf was processed by adding hydrogen atoms and termini caps with the Protein Preparation Wizard tool of the Maestro Suite [34]. Loop residues and the side chains of the modified amino acids were submitted to a minimization with OPLS3e force field [35] implemented in MacroModel 12.0 [36], using the Polak-Ribière conjugate gradient method [37] to a convergence threshold of 0.05 kJ·mol^−1^·Å^−1^ to relieve steric and electrostatic clashes. The orientation of thiol and hydroxyl groups and the conformation of asparagine, glutamine, and histidine residues were adjusted to optimize the overall hydrogen bonding network. Basic and acid amino acids were modelled in their charged state. The final structure was energy minimized with the OPLS3e force field through a first minimization run with constrained heavy atoms and a second minimization run with heavy atom positions restrained to an RMSD value of 0.3 Å.

#### 3.3.2. Ligand Docking and MM-GBSA Calculations

(*S*)-**10** and (*R*)-**10** structures were built in Maestro 11.6 [38] and minimized with the OPLS3e force field implemented in MacroModel 12.0 in implicit water [39] to an energy gradient of 0.01 kJ·mol^−1^·Å^−1^. The prepared complex between the MT_2_ receptor and 2-phenylmelatonin was used to run docking simulations. The docking grid was generated by imposing a cubic bounding box of 10 Å and an enclosing box of 20 Å, centered on 2-phenylmelatonin. After ligand removal, docking runs were performed with Glide 7.9 [40] in standard precision mode, setting MAXKEEP and MAXREF parameters, which control the number of poses to retain after the rough scoring stage and the number of poses to refine, to 50,000 and 4000, respectively. Ten poses were generated for each ligand and the pose with best GScore for each enantiomer was merged with the receptor structure. The resulting complexes were submitted to MM-GBSA calculations with Prime 5.2 implemented in the Schrodinger package [41]. The complexes were energy minimized in the GBSA continuum model to a gradient of 0.1 kJ·mol^−1^·Å^−1^ allowing the relaxation of the ligand and of receptor side chains comprised in a sphere of 12 Å from the ligand. The interaction energy was calculated subtracting the OPLS3e energy of the free protein and of the free ligand to that of the receptor-ligand complex, keeping into account for each the GBSA correction solvation term.

### 3.4. Molecular Dynamics (MD) Simulations

#### 3.4.1. MD Simulations of Ligand-Receptor Complexes

The best-ranked pose of compounds (*S*)-**10** and (*R*)-**10** was merged into the protein structure, and the complexes were energy-minimized with MacroModel 12.0 to an energy gradient of 0.01 kJ·mol^−1^·Å^−1^. During energy minimization, the ligand and residue side chains within a sphere of 5 Å were free to move, while the backbone was kept fixed. The energy minimized complexes were embedded in a pre-equilibrated POPC bilayer [42] with OPM orientation [43], setting the solute molecules distant at least 13 Å from the periodic neighboring images and solvated with TIP3P water molecules. System net charge was neutralized by adding 10 Cl^-^ ions. Molecular dynamics simulations were carried out for 150 ns on the equilibrated system (the detailed equilibration protocol is reported in Protocol S1 in Supplementary Materials) in NPT ensemble at 1.0 atm by applying a Langevin coupling scheme [44] with a relaxation time of 1.0 ps at 300 K and with a damping coefficient of 2.0 ps^−1^. Alpha carbons were restrained with 0.1 kcal·mol^−1^·Å^−2^, while the backbone heavy atoms of the capped terminal residues were restrained with a force constant of 1.0 kcal·mol^−1^·Å^−2^.

#### 3.4.2. MD Simulations of Melatoninergic Ligands in Solution

MD simulations were performed by solvating the compounds in a cubic solvent box of chloroform of 8 × 8 × 8 Å comprising 2560 molecules. The box was modelled in Maestro 11.6 applying the OPLS3e force field and equilibrated by performing 1.5-ns-long MD simulation at 300 K in NPT ensemble. The equilibrated box was then used to solvate the ligand setting the minimum distance from any solute atom to 12 Å in the x, y, and z direction. The system was equilibrated using the default equilibration protocol implemented in Desmond 5.4 [45] within Schrodinger 2018–2 suite. The equilibration was followed by a production stage of 2 μs at 300 K in NVT ensemble with Langevin thermostat coupled with a relaxation time of 1 ps. Forty-thousand snapshots were collected during the simulation and projected onto a binned τ_1_–τ_2_ 2D plot, and a relative free energy value was calculated for each bin *i* as:

(1) $$G_{i}=-RT\ln\left(N_{i}/N_{0}\right)$$

where $N_{i}$ and $N_{0}$ are the number of snapshots contained in the bin i and in the most populated bin, respectively. τ_1_ and τ_2_ are defined in Figure 3.

The convergence was assessed by evaluating the evolution of the free-energy surface during MD simulation. We considered the simulation in chloroform to be converged as the free-energy profiles did not change significantly in the last 1 μs of simulation (Figure S21 in Supplementary Materials).

The same protocol was applied to ligands (*i*) solvated in a TIP3P water box and (*ii*) embedded in a POPC bilayer solvated in TIP3P water molecules. The simulation in the TIP3P water box was conducted for 2 µs, collecting 40,000 snapshots for free-energy surface calculation. The simulation in the membrane system lasted for 4 µs, for a total of 80,000 collected snapshots, due to the longer time required for the convergence of the free-energy surface.

#### 3.4.3. MD Simulation General Procedures

Simulations (ligand-receptor complexes and solvated ligands) were conducted with Desmond 5.4 with the OPLS3e force field. Bond lengths to hydrogen atoms were constrained by applying the M-SHAKE algorithm [46]. Short-range electrostatic interactions were cut-off at 9 Å, whereas long-range electrostatic interactions were treated using the smooth Particle Mesh Ewald method [47]. A RESPA integrator [48] was used with a time-step of 2 fs, while long-range electrostatic interactions were computed every 6 fs.

## 4. Conclusions

The introduction of a beta-methyl group in the ethylamide side chain of the melatonin bioisostere UCM793 (compound **3**) led to a couple of enantiomers characterized by a stereoselective behavior, with the eutomer having higher binding affinity than the unsubstituted parent compound. Docking studies into the melatonin receptor crystal structure coupled to evaluation of conformational equilibria in solution led to the conclusion that the beta-methyl group generated an enrichment of the bioactive conformation assumed by the eutomer bound to the melatonin receptor. Docking studies also identified a binding site for the methyl group, which might be further explored and exploited for the synthesis of new ligands.

The compounds here described do not show clear advantages over known ligands, being slightly less potent than melatonin itself, and their safety and pharmacokinetics are unknown. On the other hand, they belong to a versatile chemical class that includes compounds, which had shown interesting in vivo activities [15,16]. Moreover, this study shows that it is possible to improve melatoninergic activity modulating the conformational space and occupying a pocket within the melatonin receptors, which has not been discussed so far.

These results represent a clear example of the interplay between chirality and conformational equilibria, which should be taken into account when stereoselectivity data are analyzed to devise structure-activity relationships. In the particular case of melatoninergic ligands, the conformational effect of the beta-methyl group, here thoroughly investigated for *N*-anilinoethylamides, also applies to melatonin-like indole derivatives, confirming the assessed bioisosterism between the indole and the anilino scaffolds.

## Figures and Tables

**Table 1:**
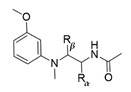
Binding affinity and intrinsic activity of alpha- and beta-methyl *N*-anilinoethylamides for human MT_1_ and MT_2_ melatonin receptors.

Compound	R_α_	R_β_	hMT_1_		hMT_2_	
			p*K*_i_ (±SD) *^a^*	IA (±SD) *^b^*	p*K*_i_ (±SD) *^b^*	IA (±SD) *^b^*
melatonin	--	--	9.60 ± 0.18	1.00 ± 0.09	9.44 ± 0.12	1.00 ± 0.07
**3**	H	H	8.76 ± 0.18	0.95 *^c^*	8.65 ± 0.01	1.06 *^c^*
**10**	H	Me	8.67 ± 0.01	0.98 ± 0.06	8.77 ± 0.02	1.01 ± 0.07
(*R*)-**10** *^d^*	H	Me	7.59 ± 0.02	1.00 ± 0.07	7.91 ± 0.04	0.99 ± 0.08
(*S*)-**10** *^e^*	H	Me	8.88 ± 0.08	0.98 ± 0.11	8.96 ± 0.11	1.01 ± 0.10
**12**	Me	H	8.17 ± 0.02	n.d.	7.98 ± 0.08	n.d.

*^a^* p*Ki* values were calculated from IC_50_ values, obtained from competition curves by the method of Cheng and Prusoff [21], and are the mean of at least three determinations performed in duplicate. *^b^* The relative intrinsic activity values were obtained by dividing the maximum analogue-induced G-protein activation by that of melatonin Measurements were performed in triplicate. *^c^* Ref. [18]. *^d^* Enantiomeric excess: 95%. *^e^* Enantiomeric excess: 81%. n.d. not determined.

## Supplementary Materials

These data are available online. Figures S1–S13. ^1^H NMR spectra of compounds **6**, **7**, **8**, **9**, **10** and **12**, ^13^C NMR spectra of compounds **6**, **7**, **9**, **10** and **12**, NOESY spectrum of compound **10** and COSY spectrum of compound **12**. Figures S14–S19. Chiral HPLC chromatograms of compounds 6, (*R*)-6, (*S*)-6, 10, (*R*)-10 and (*S*)-10. Figure S20. Free-energy surfaces calculated from the MD simulation of (*S*)-10 in a POPC membrane model and in TIP3P water molecules. Protocol S1. Equilibration protocol for MD simulations of MT_2_ receptor-ligand complexes. Figure S21. Time-evolution of the free energy surface calculated from the MD simulation of (*S*)-10 in explicit chloroform.

## References

[1] Hardeland R., Cardinali D.P., Srinivasan V., Spence D.W., Brown G.M., Pandi-Perumal S.R. (2011). Melatonin—A pleiotropic, orchestrating regulator molecule. Prog. Neurobiol..

[2] Jockers R., Delagrange P., Dubocovich M.L., Markus R.P., Renault N., Tosini G., Cecon E., Zlotos D.P. (2016). Update on melatonin receptors: IUPHAR Review 20: Melatonin receptors. Br. J. Pharmacol..

[3] Cecon E., Oishi A., Jockers R. (2018). Melatonin receptors: Molecular pharmacology and signalling in the context of system bias. Br. J. Pharmacol..

[4] Liu J., Clough S.J., Hutchinson A.J., Adamah-Biassi E.B., Popovska-Gorevski M., Dubocovich M.L. (2016). MT_1_ and MT_2_ melatonin receptors: A therapeutic perspective. Annu. Rev. Pharmacol. Toxicol..

[5] Zlotos D.P., Jockers R., Cecon E., Rivara S., Witt-Enderby P.A. (2014). MT_1_ and MT_2_ melatonin receptors: Ligands, Models, oligomers, and therapeutic potential. J. Med. Chem..

[6] Boutin J.A., Witt-Enderly P.A., Sotriffer C., Zlotos D.P. (2020). Melatonin receptor ligands: A pharmaco-chemical perspective. J. Pineal Res..

[7] Mulchahey J., Goldwater D., Zemlan F. (2004). A single blind, placebo controlled, across groups dose escalation study of the safety, tolerability, pharmacokinetics and pharmacodynamics of the melatonin analog β-methyl-6-chloromelatonin. Life Sci..

[8] Ettaoussi M., Sabaouni A., Pérès B., Landagaray E., Nosjean O., Boutin J.A., Caignard D.-H., Delagrange P., Berthelot P., Yous S. (2013). Synthesis and pharmacological evaluation of a series of the agomelatine analogues as melatonin MT_1_ /MT_2_ agonist and 5-HT_2C_ antagonist. ChemMedChem.

[9] Ettaoussi M., Pérès B., Jarry C., Nosjean O., Boutin J.A., Gohier A., Mannoury la Cour C., Caignard D.-H., Delagrange P., Berthelot P. (2014). Synthesis, chiral resolution, absolute configuration assignment and pharmacological evaluation of a series of melatoninergic ligands. Med. Chem. Commun..

[10] Carocci A., Catalano A., Lovece A., Lentini G., Duranti A., Lucini V., Pannacci M., Scaglione F., Franchini C. (2010). Design, synthesis, and pharmacological effects of structurally simple ligands for MT_1_ and MT_2_ melatonin receptors. Bioorg. Med. Chem..

[11] Rivara S., Diamantini G., Di Giacomo B., Lamba D., Gatti G., Lucini V., Pannacci M., Mor M., Spadoni G., Tarzia G. (2006). Reassessing the melatonin pharmacophore-enantiomeric resolution, pharmacological activity, structure analysis, and molecular modeling of a constrained chiral melatonin analogue. Bioorg. Med. Chem..

[12] Bedini A., Lucarini S., Spadoni G., Tarzia G., Scaglione F., Dugnani S., Pannacci M., Lucini V., Carmi C., Pala D. (2011). Toward the definition of stereochemical requirements for MT_2_-selective antagonists and partial agonists by studying 4-phenyl-2-propionamidotetralin derivatives. J. Med. Chem..

[13] Stauch B., Johansson L.C., McCorvy J.D., Patel N., Han G.W., Huang X.-P., Gati C., Batyuk A., Slocum S.T., Ishchenko A. (2019). Structural basis of ligand recognition at the human MT_1_ melatonin receptor. Nature.

[14] Johansson L.C., Stauch B., McCorvy J.D., Han G.W., Patel N., Huang X.-P., Batyuk A., Gati C., Slocum S.T., Li C. (2019). XFEL structures of the human MT_2_ melatonin receptor reveal the basis of subtype selectivity. Nature.

[15] Ochoa-Sanchez R., Comai S., Lacoste B., Bambico F.R., Dominguez-Lopez S., Spadoni G., Rivara S., Bedini A., Angeloni D., Fraschini F. (2011). Promotion of Non-Rapid Eye Movement Sleep and Activation of Reticular Thalamic Neurons by a Novel MT2 Melatonin Receptor Ligand. J. Neurosci..

[16] Lopez-Canul M., Palazzo E., Dominguez-Lopez S., Luongo L., Lacoste B., Comai S., Angeloni D., Fraschini F., Boccella S., Spadoni G. (2015). Selective melatonin MT2 receptor ligands relieve neuropathic pain through modulation of brainstem descending antinociceptive pathways. Pain.

[17] Rivara S., Vacondio F., Fioni A., Silva C., Carmi C., Mor M., Lucini V., Pannacci M., Caronno A., Scaglione F. (2009). *N*-(Anilinoethyl)amides: Design and synthesis of metabolically stable, selective melatonin receptor ligands. ChemMedChem.

[18] Rivara S., Lodola A., Mor M., Bedini A., Spadoni G., Lucini V., Pannacci M., Fraschini F., Scaglione F., Sanchez R.O. (2007). *N*-(Substituted-anilinoethyl) amides: Design, synthesis, and pharmacological characterization of a new class of melatonin receptor ligands. J. Med. Chem..

[19] Etxabe J., Izquierdo J., Landa A., Oiarbide M., Palomo C. (2015). Catalytic enantioselective synthesis of N,C^α^,C^α^-trisubstituted α-amino acid derivatives using 1*H* -imidazol-4(5*H* )-ones as key templates. Angew. Chem. Int. Ed..

[20] Bartoccini F., Venturi S., Retini M., Mari M., Piersanti G. (2019). Total synthesis of (−)-clavicipitic acid via γ,γ-dimethylallyltryptophan (DMAT) and chemoselective C–H hydroxylation. J. Org. Chem..

[21] Cheng Y., Prusoff W.H. (1973). Relationship between the inhibition constant (Ki) and the concentration of inhibitor which causes 50% inhibition (I_50_) of an enzymatic reaction. Biochem. Pharmacol..

[22] Ballesteros J.A., Weinstein H. (1995). Integrated methods for the construction of three-dimensional models and computational probing of structure-function relations in G protein-coupled receptors. Methods Neurosci..

[23] Hassan Mohamed I., Giorgio C., Incerti M., Russo S., Pala D., Pasquale E.B., Zanotti I., Vicini P., Barocelli E., Rivara S. (2014). UniPR 129 is a competitive small molecule Eph-ephrin antagonist blocking in vitro angiogenesis at low micromolar concentrations. Br. J. Pharmacol..

[24] Lu H., Martí J. (2019). Binding and dynamics of melatonin at the interface of phosphatidylcholine-cholesterol membranes. PLoS ONE.

[25] Lu H., Martí J. (2020). Cellular absorption of small molecules: Free energy landscapes of melatonin binding at phospholipid membranes. Sci. Rep..

[26] Lukas R.J., Muresan A.Z., Damaj M.I., Blough B.E., Huang X., Navarro H.A., Mascarella S.W., Eaton J.B., Marxer-Miller S.K., Carroll F.I. (2010). Synthesis and characterization of in vitro and in vivo profiles of hydroxybupropion analogues: Aids to smoking cessation. J. Med. Chem..

[27] Chattopadhyay A.K., Ly V.L., Jakkepally S., Berger G., Hanessian S. (2016). Total Synthesis of Isodaphlongamine H: A Possible Biogenetic Conundrum. Angew. Chem. Int. Ed..

[28] Nonno R., Lucini V., Pannacci M., Mazzucchelli C., Angeloni D., Fraschini F., Stankov B.M. (1998). Pharmacological characterization of the human melatonin Mel1a receptor following stable transfection into NIH3T3 cells. Br. J. Pharmacol..

[29] Spadoni G., Balsamini C., Bedini A., Diamantini G., Di Giacomo B., Tontini A., Tarzia G., Mor M., Plazzi P.V., Rivara S. (1998). 2-[N-Acylamino(C1-C3)alkyl]indoles as MT_1_ melatonin receptor partial agonists, antagonists, and putative inverse agonists. J. Med. Chem..

[30] Bradford M.M. (1976). A rapid and sensitive method for the quantitation of microgram quantities of protein utilizing the principle of protein−dye binding. Anal. Biochem..

[31] Wallez V., Durieux-Poissonnier S., Chavatte P., Boutin J.A., Audinot V., Nicolas J.P., Bennejean C., Delagrange P., Renard P., Lesieur D. (2002). Synthesis and structure−affinity-activity relationships of novel benzofuran derivatives as MT(2) melatonin receptor selective ligands. J. Med. Chem..

[32] Fiser A., Do R.K.G., Šali A. (2000). Modeling of loops in protein structures. Protein Sci..

[33] Shen M., Šali A. (2006). Statistical potential for assessment and prediction of protein structures. Protein Sci..

[34] (2016). Schrödinger Release 2018-2: Protein Preparation Wizard.

[35] Roos K., Wu C., Damm W., Reboul M., Stevenson J.M., Lu C., Dahlgren M.K., Mondal S., Chen W., Wang L. (2019). OPLS3e: Extending force field coverage for drug-like small molecules. J. Chem. Theory Comput..

[36] (2018). Schrödinger Release 2018-2: MacroModel 12.0.

[37] Polak E., Ribière G. (1969). Note sur la convergence de méthodes de directions conjuguées. ESAIM Math. Model. Numer. Anal. Modél. Math. Anal. Numér..

[38] (2018). Schrödinger Release 2018-2: Maestro 11.6.

[39] Still W.C., Tempczyk A., Hawley R.C., Hendrickson T. (1990). Semianalytical treatment of solvation for molecular mechanics and dynamics. J. Am. Chem. Soc..

[40] Friesner R.A., Banks J.L., Murphy R.B., Halgren T.A., Klicic J.J., Mainz D.T., Repasky M.P., Knoll E.H., Shelley M., Perry J.K. (2004). Glide: A new approach for rapid, accurate docking and scoring. 1. method and assessment of docking accuracy. J. Med. Chem..

[41] (2018). Schrödinger Release 2018-2: Prime 5.2.

[42] Klauda J.B., Venable R.M., Freites J.A., O’Connor J.W., Tobias D.J., Mondragon-Ramirez C., Vorobyov I., MacKerell A.D., Pastor R.W. (2010). Update of the CHARMM all-atom Additive force field for lipids: Validation on six lipid types. J. Phys. Chem. B.

[43] Lomize M.A., Pogozheva I.D., Joo H., Mosberg H.I., Lomize A.L. (2012). OPM database and PPM web server: Resources for positioning of proteins in membranes. Nucleic Acids Res..

[44] Feller S.E., Zhang Y., Pastor R.W., Brooks B.R. (1995). Constant pressure molecular dynamics simulation: The Langevin piston method. J. Chem. Phys..

[45] (2018). Schrödinger Release 2018-2: Desmond Molecular Dynamics System 5.4.

[46] Kräutler V., Van Gunsteren W.F., Hünenberger P.H. (2001). A fast SHAKE algorithm to solve distance constraint equations for small molecules in molecular dynamics simulations. J. Comput. Chem..

[47] Essmann U., Perera L., Berkowitz M.L., Darden T., Lee H., Pedersen L.G. (1995). A smooth particle mesh Ewald method. J. Chem. Phys..

[48] Tuckerman M., Berne B.J., Martyna G.J. (1992). Reversible multiple time scale molecular dynamics. J. Chem. Phys..

