# Supplementary material for: Chiral Recognition of Flexible Melatonin Receptor Ligands Induced by Conformational Equilibria
Source: Molecules. 2020 Sep 4;25(18):4057. doi: 10.3390/molecules25184057 (PMC7570888; doi:10.3390/molecules25184057)

Chiral recognition of flexible melatonin receptor ligands induced by conformational equilibria

Gian Marco Elisi [1,^], Annalida Bedini [2,^], Laura Scalvini [1], Caterina Carmi [1], Silvia Bartolucci [2], Valeria Lucini [3], Francesco Scaglione [3], Marco Mor [1,*], Silvia Rivara [1], Gilberto Spadoni [2]

[1] Dipartimento di Scienze degli Alimenti e del Farmaco, Università degli Studi di Parma, Parco Area delle Scienze 27/A, I-43124 Parma, Italy; E-mails: gianmarco.elisi@unipr.it; laura.scalvini@unipr.it; caterina.carmi@yahoo.it; silvia.rivara@unipr.it

[2] Dipartimento di Scienze Biomolecolari, Università degli Studi di Urbino "Carlo Bo", Piazza Rinascimento 6, I-61029 Urbino, Italy; E-mails: annalida.bedini@uniurb.it; silvia.bartolucci@uniurb.it; gilberto.spadoni@uniurb.it.

[3] Dipartimento di Oncologia ed Emato-oncologia, Università degli Studi di Milano, Via Vanvitelli 32, I-20129 Milano, Italy; E-mails: valeria.lucini@unimi.it; francesco.scaglione@unimi.it

* Correspondence: marco.mor@unipr.it; Tel.: +39 0521 905059

^ These authors equally contributed to the research.

Supplementary Material

Figures S1-13. ^{1}H NMR spectra of compounds **6**, **7**, **8**, **9**, **10** and **12**, ^{13}C NMR spectra of compounds **6**, **7**, **9**, **10** and **12**, NOESY spectrum of compound **10** and COSY spectrum of compound **12**.

Figures S14-19. Chiral HPLC chromatograms of compounds **6**, **(*R*)-6**, **(*S*)-6**, **10**, **(*R*)-10** and **(*S*)-10**.

Figure S20. Free-energy surfaces calculated from the MD simulation of **(*S*)-10** in POPC membrane model and in TIP3P water molecules.

Protocol S1. Equilibration protocol for MD simulations of MT_2 receptor-ligand complexes.

Figure S21. Time-evolution of the free energy surface calculated from the MD simulation of **(*S*)-10** in chloroform.

Figure S1. ^{1}H NMR spectrum (200 MHz, $CDCl_3$) of compound **6**.

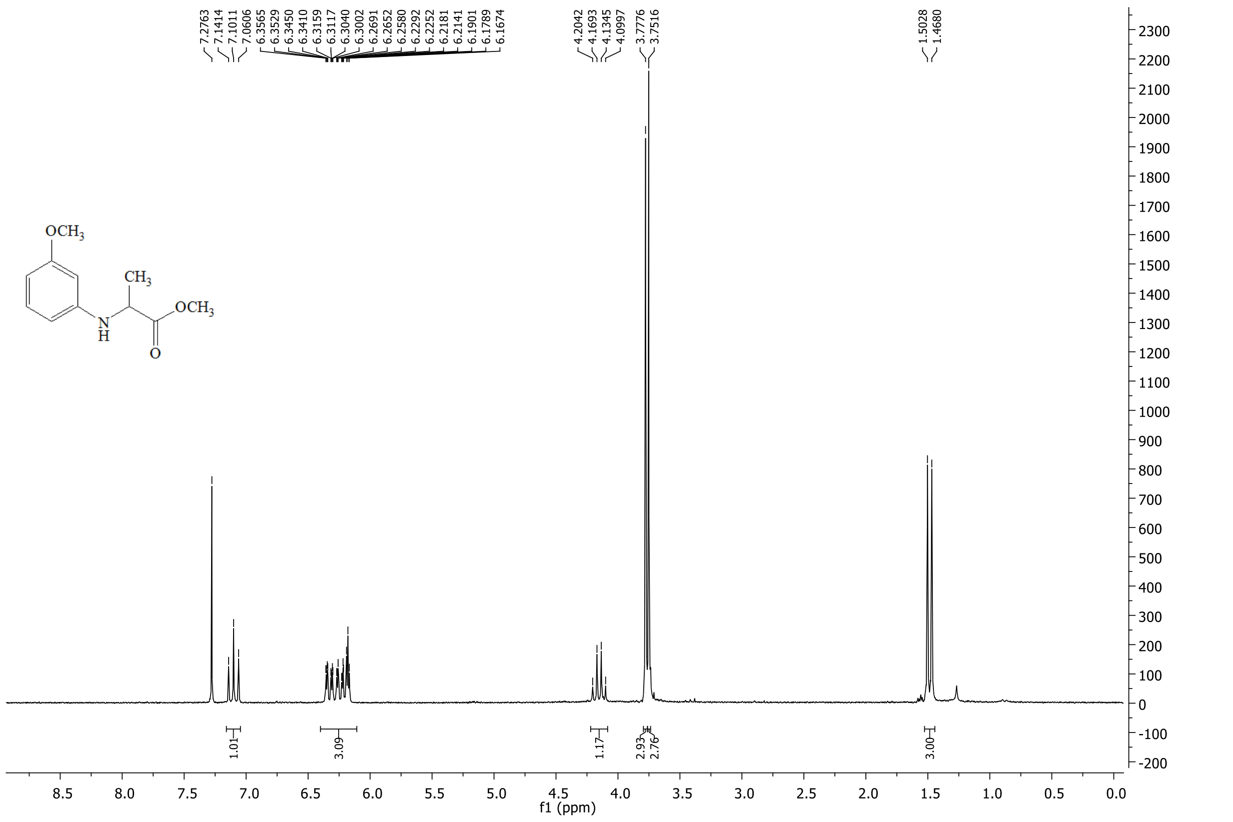

Figure S2. ^{13}C NMR spectrum (100 MHz, $CDCl_3$) of compound **6**.

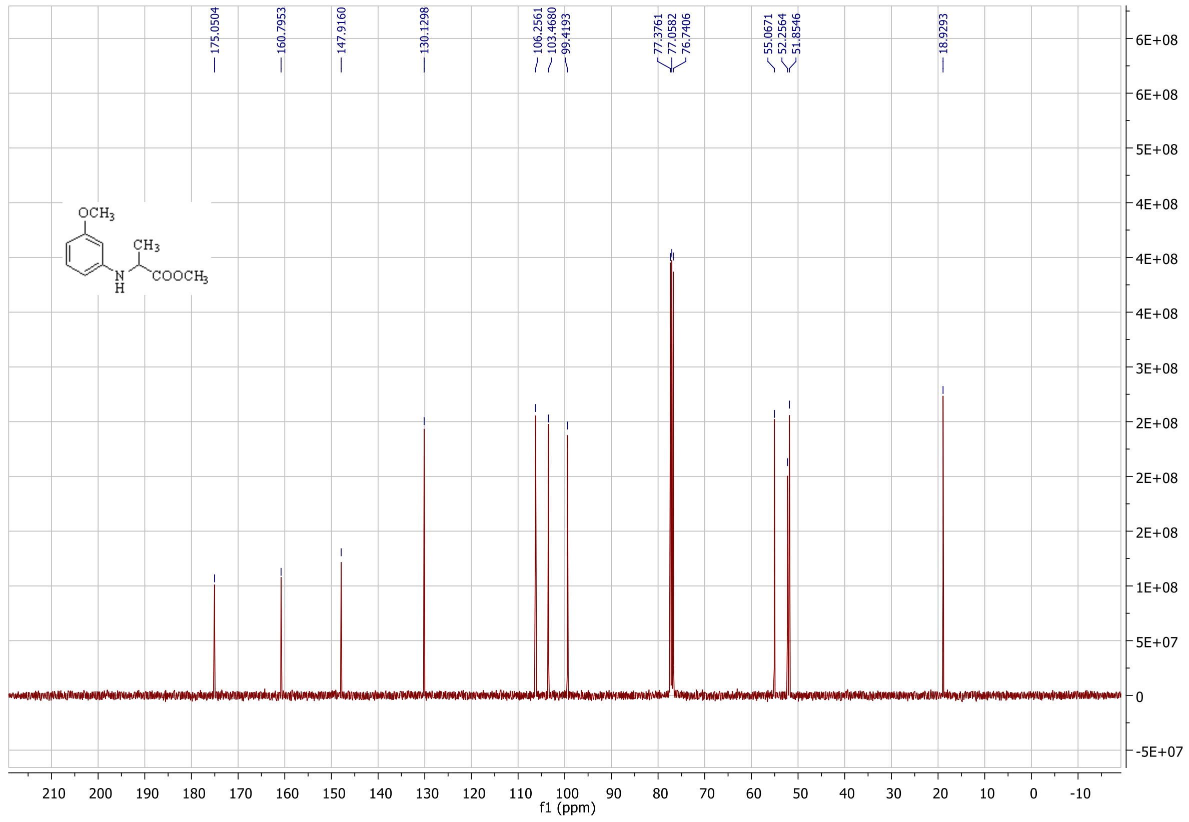

Figure S3. ^{1}H NMR spectrum (400 MHz, $CDCl_3$) of compound **7**.

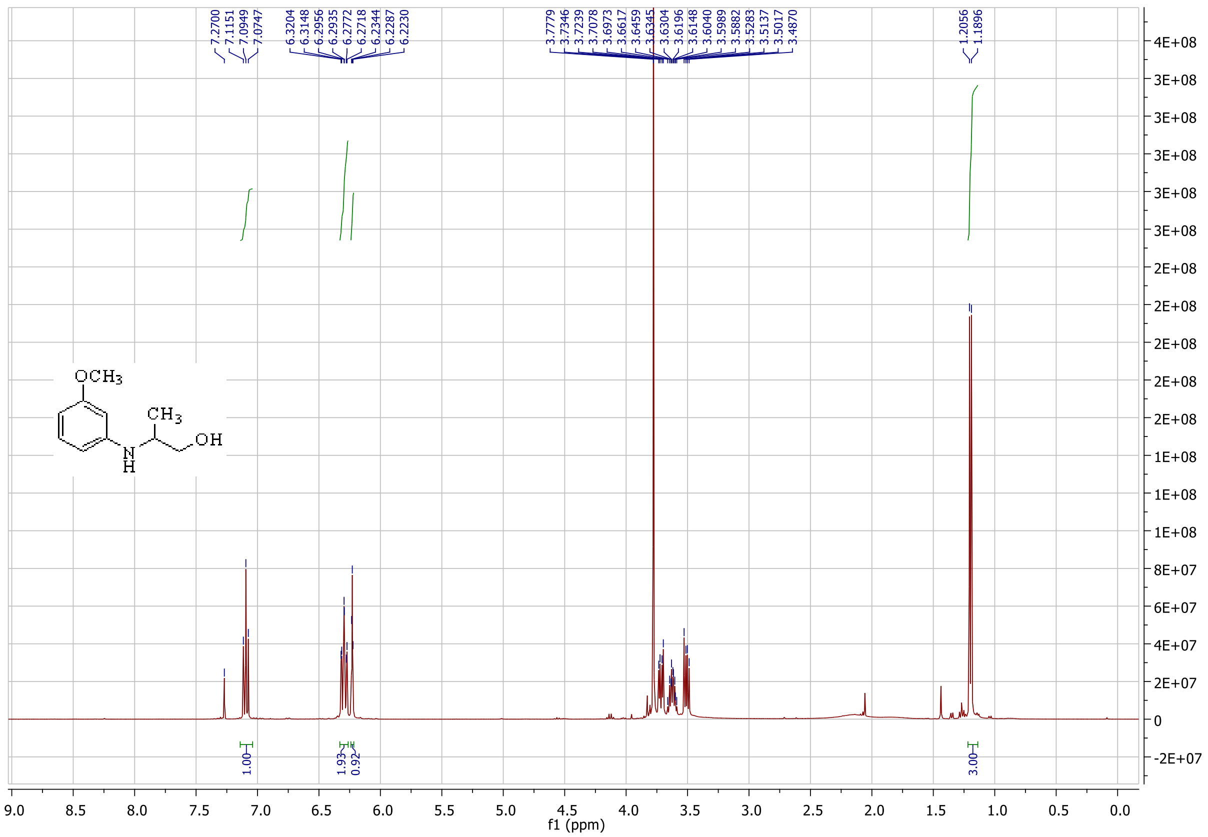

Figure S4. ^{13}C NMR spectrum (100 MHz, $CDCl_3$) of compound **7**.

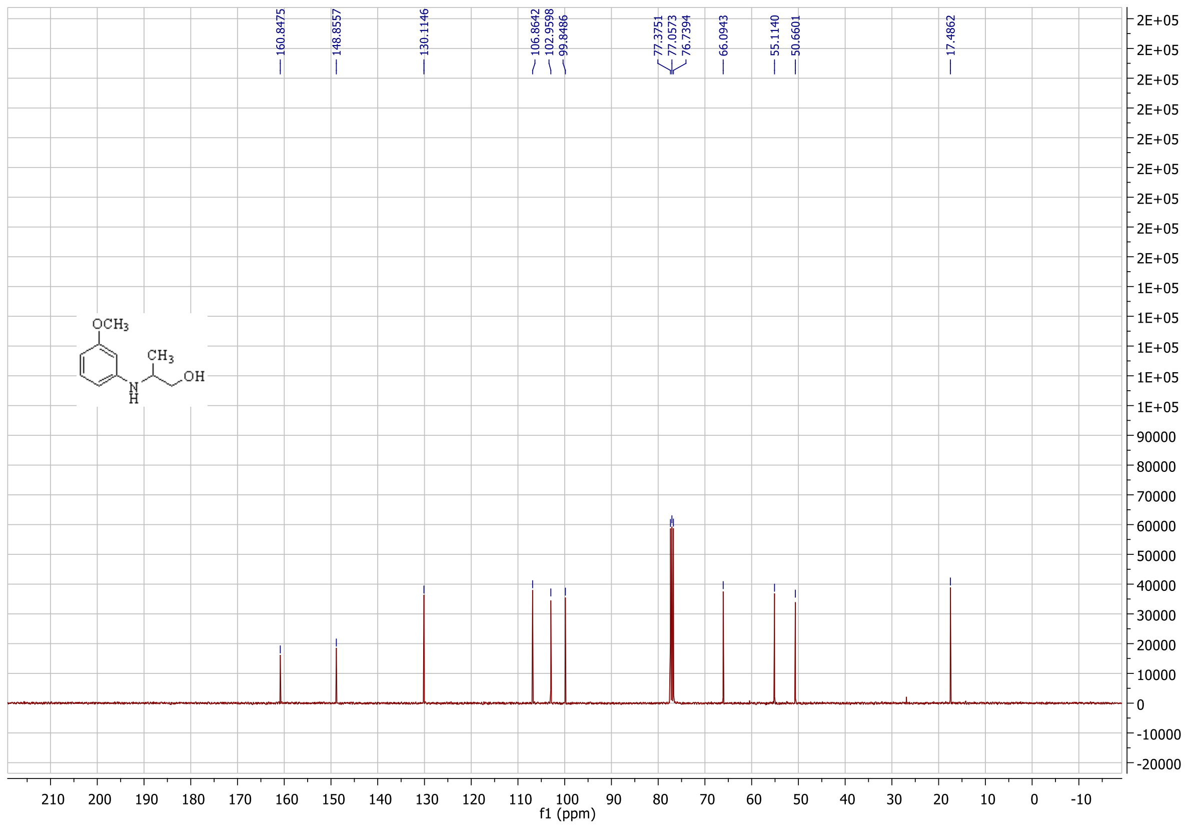

Figure S5. ^{1}H NMR spectrum (200 MHz, $CDCl_3$) of compound **8**.

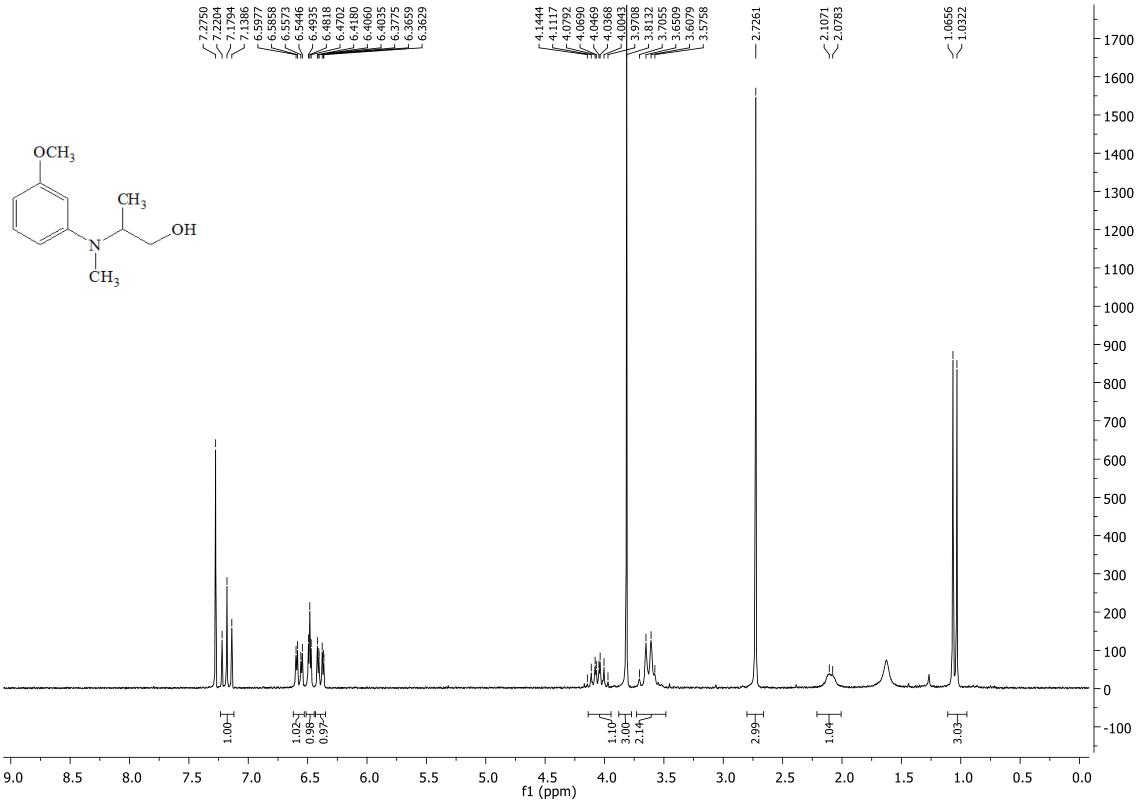

Figure S6. ^{1}H NMR spectrum (200 MHz, $CDCl_3$) of compound **9**.

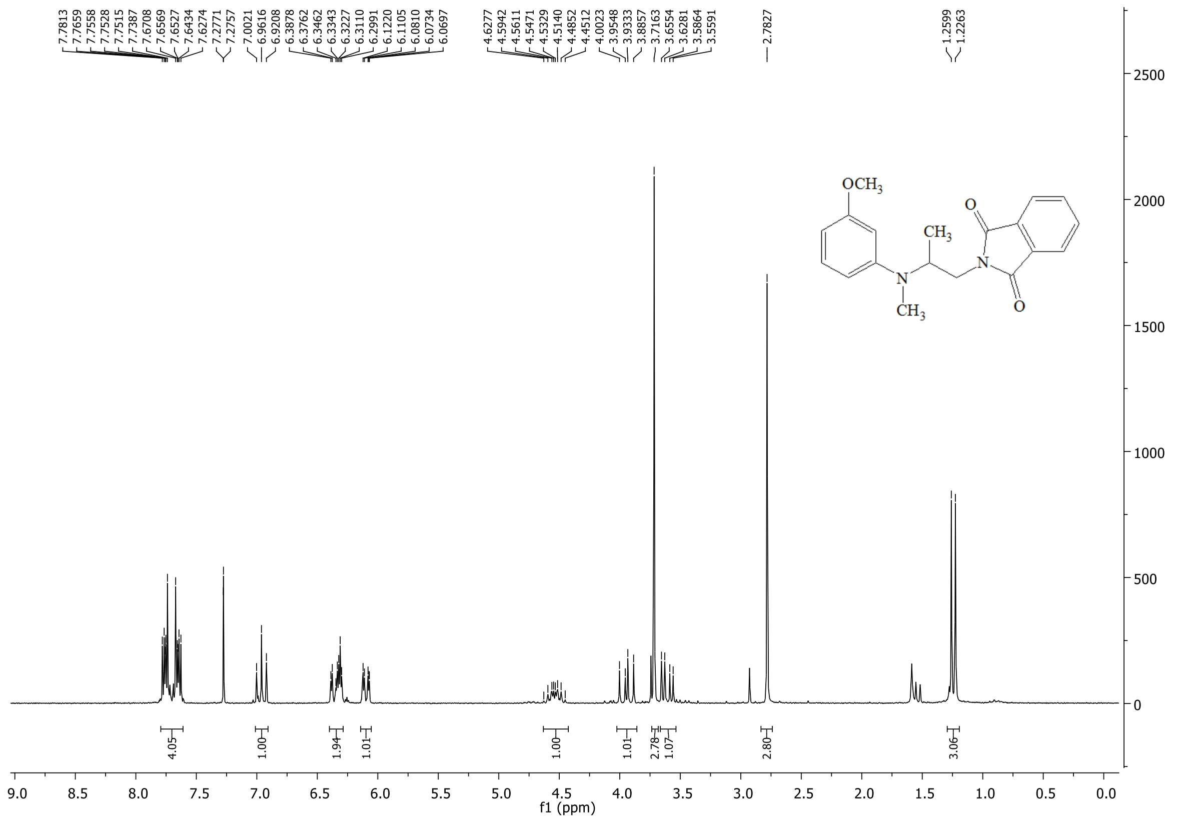

Figure S7. ^{13}C NMR spectrum (100 MHz, $CDCl_3$) of compound **9**.

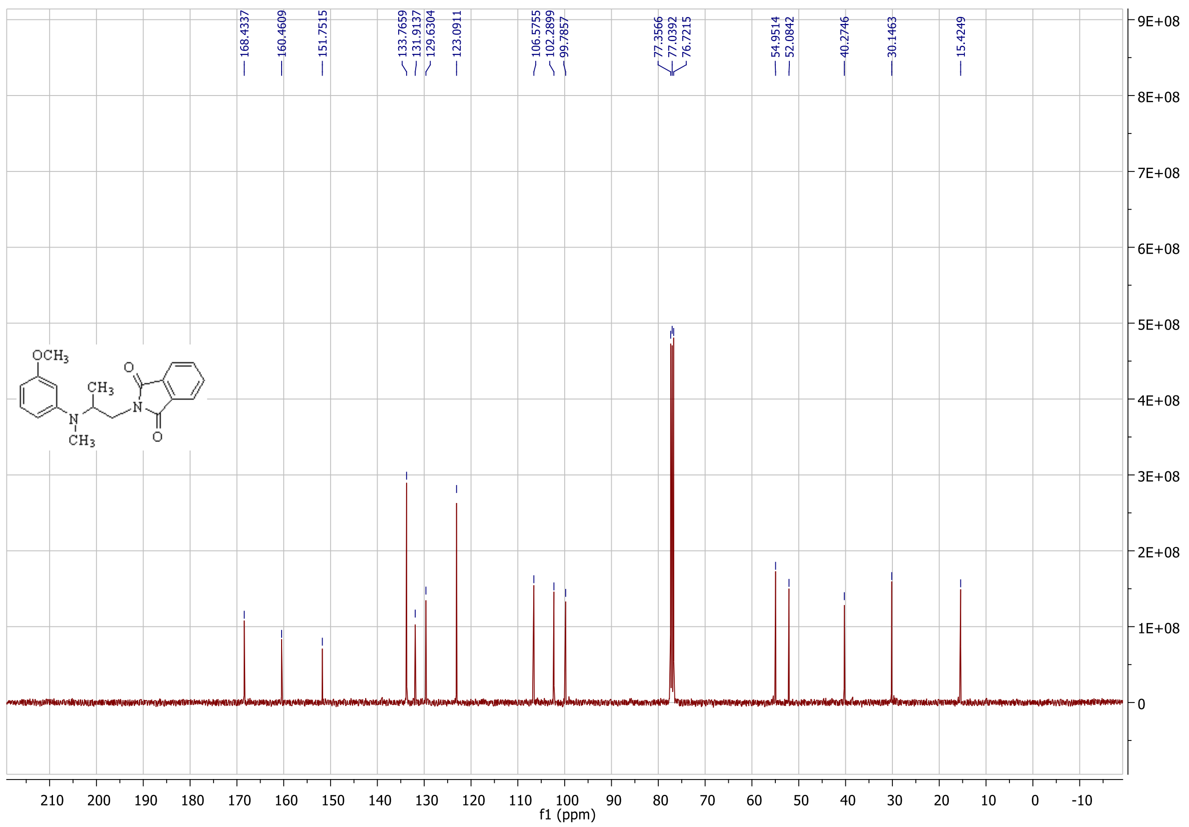

Figure S8. ^{1}H NMR spectrum (600 MHz, $CDCl_3$) of compound **10**.

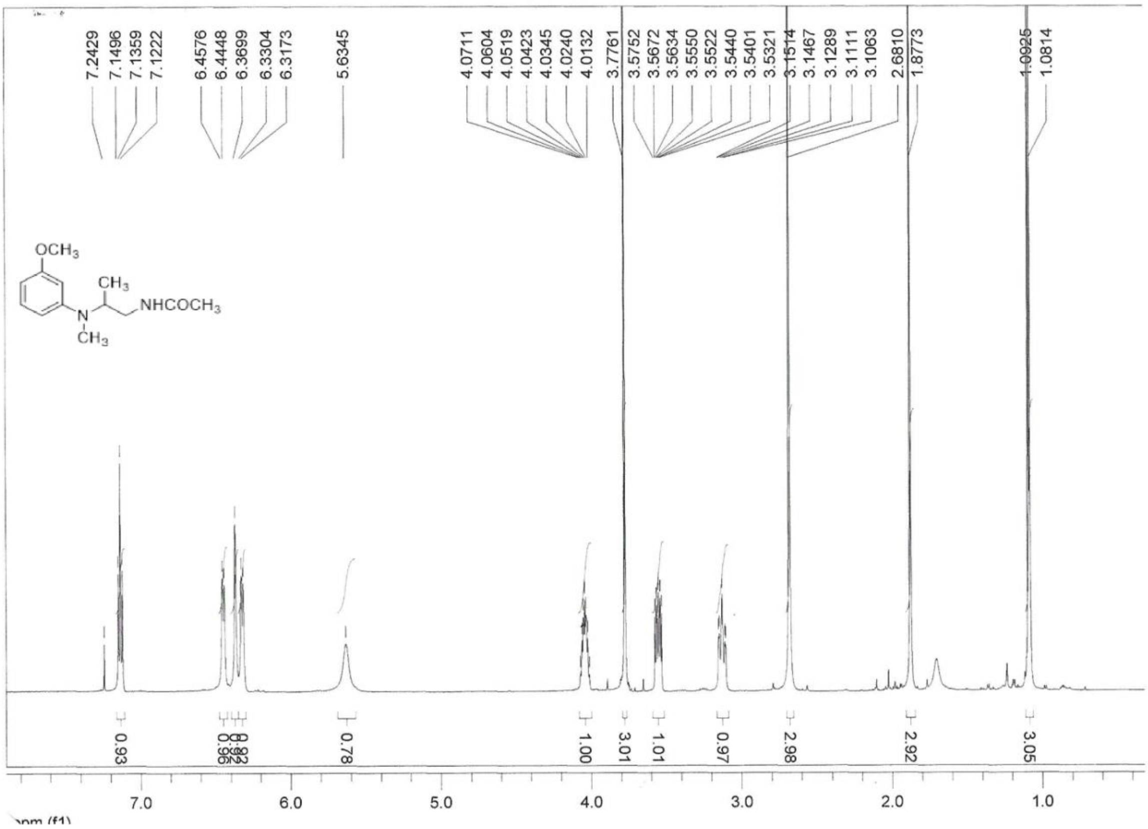

Figure S9. ^{13}C NMR spectrum (100 MHz, $CDCl_3$) of compound **10**.

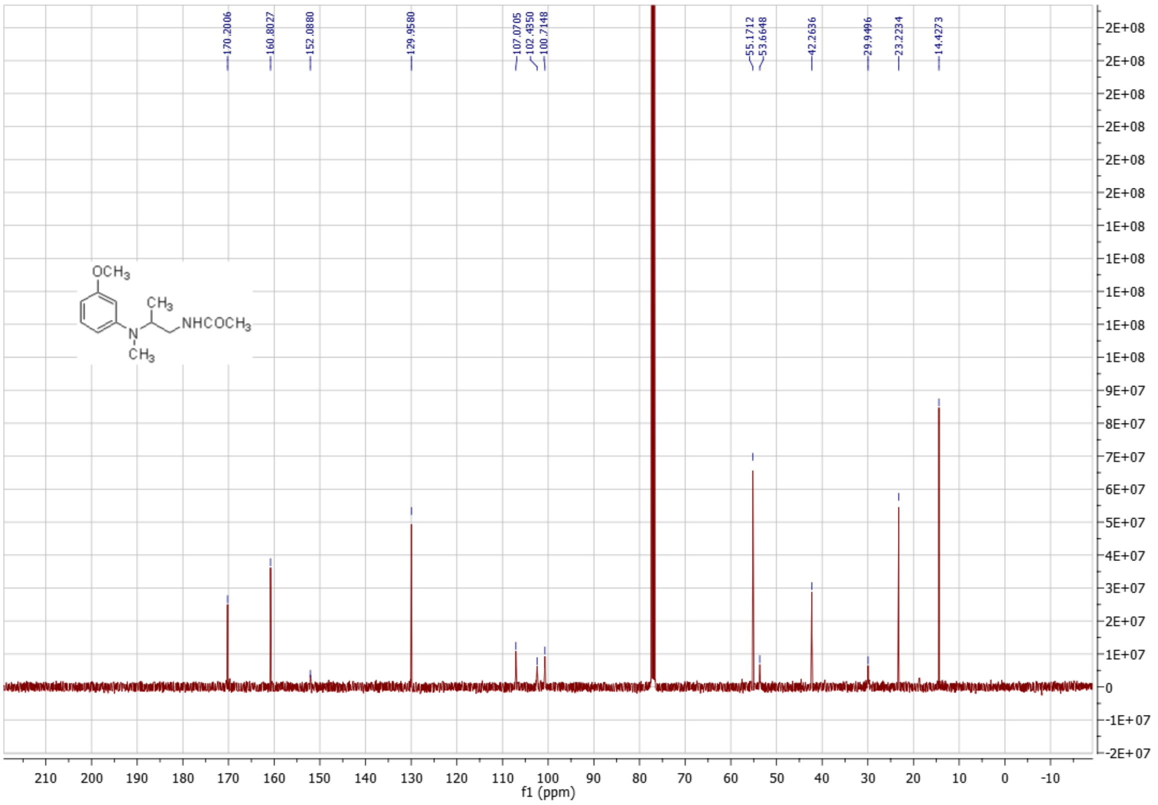

Figure S10. NOESY peaks (in red) of compound **10** (600 MHz, $CDCl_3$, 298 K). Proton assignment: (600 MHz, $CDCl_3$) δ 1.09 (CH<u>CH</u>3), 1.89 (CO<u>CH</u>3), 2.68 (N<u>CH</u>3), 3.13 (Hα2), 3.55 (Hα1), 3.77 (O<u>CH</u>3), 4.04 (Hβ), 5.63 (N<u>H</u>), 6.32 (H4), 6.37 (H2), 6.45 (H6), 7.13 (H5).

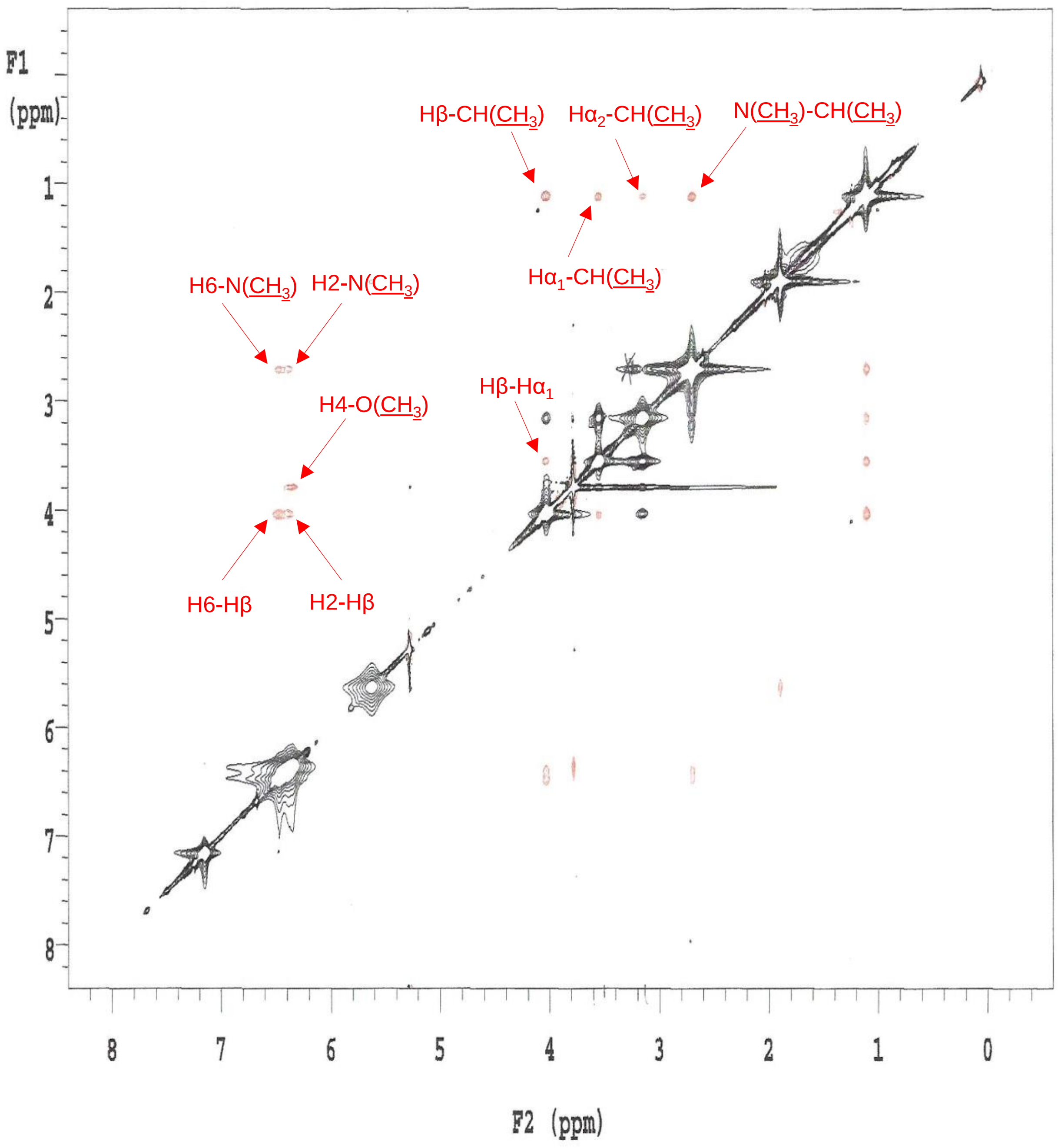

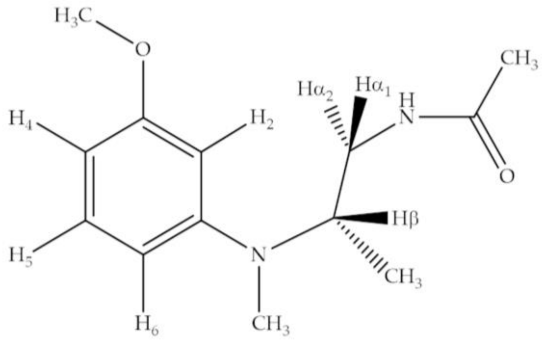

Figure S11. ^{1}H NMR spectrum (400 MHz, $CDCl_3$) of compound **12**.

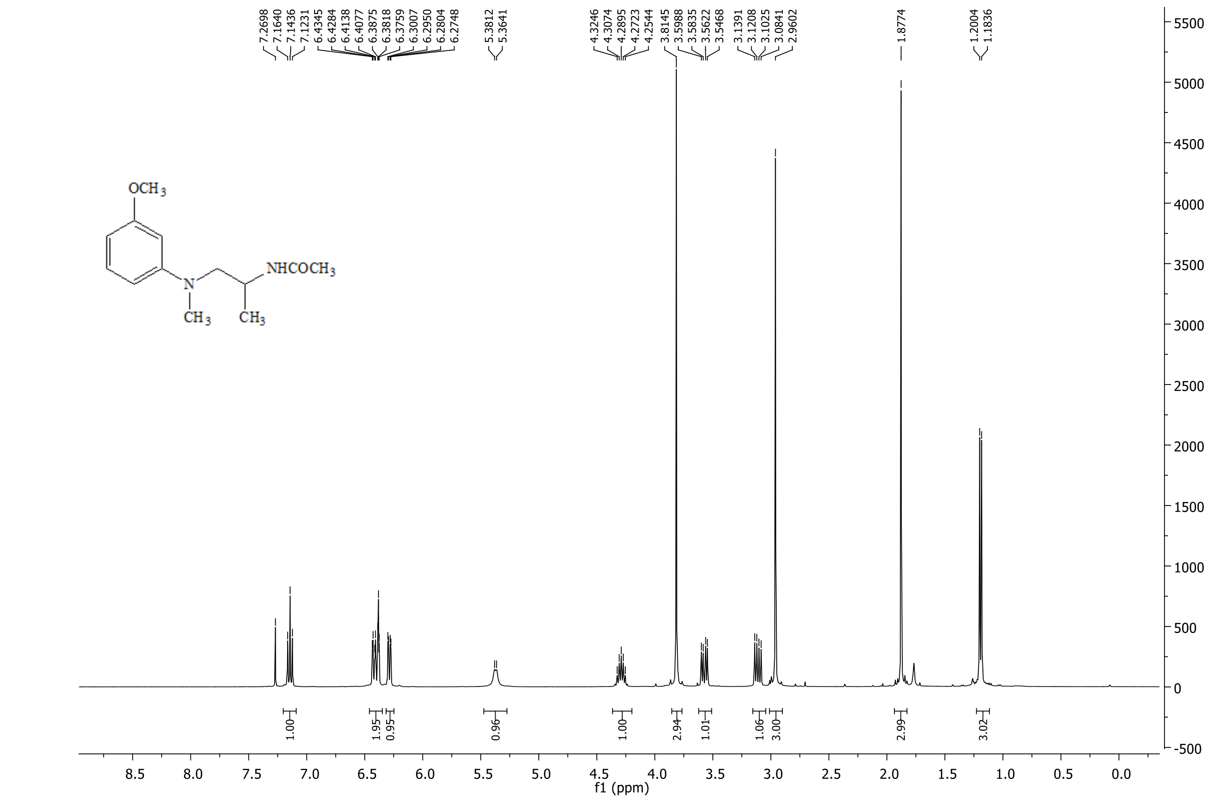

Figure S12. ^{13}C NMR spectrum (100 MHz, $CDCl_3$) of compound **12**.

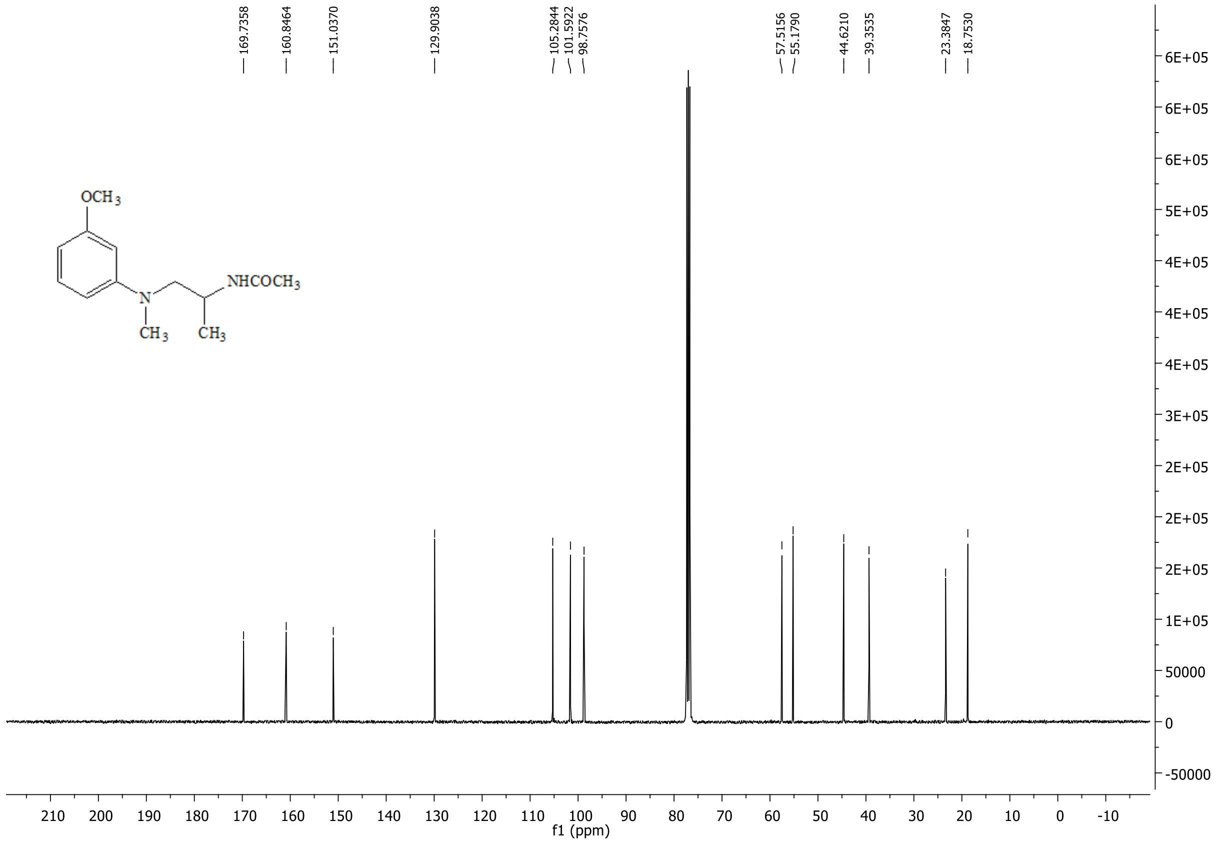

Figure S13. COSY spectrum (400 MHz, $CDCl_3$) of compound **12**.

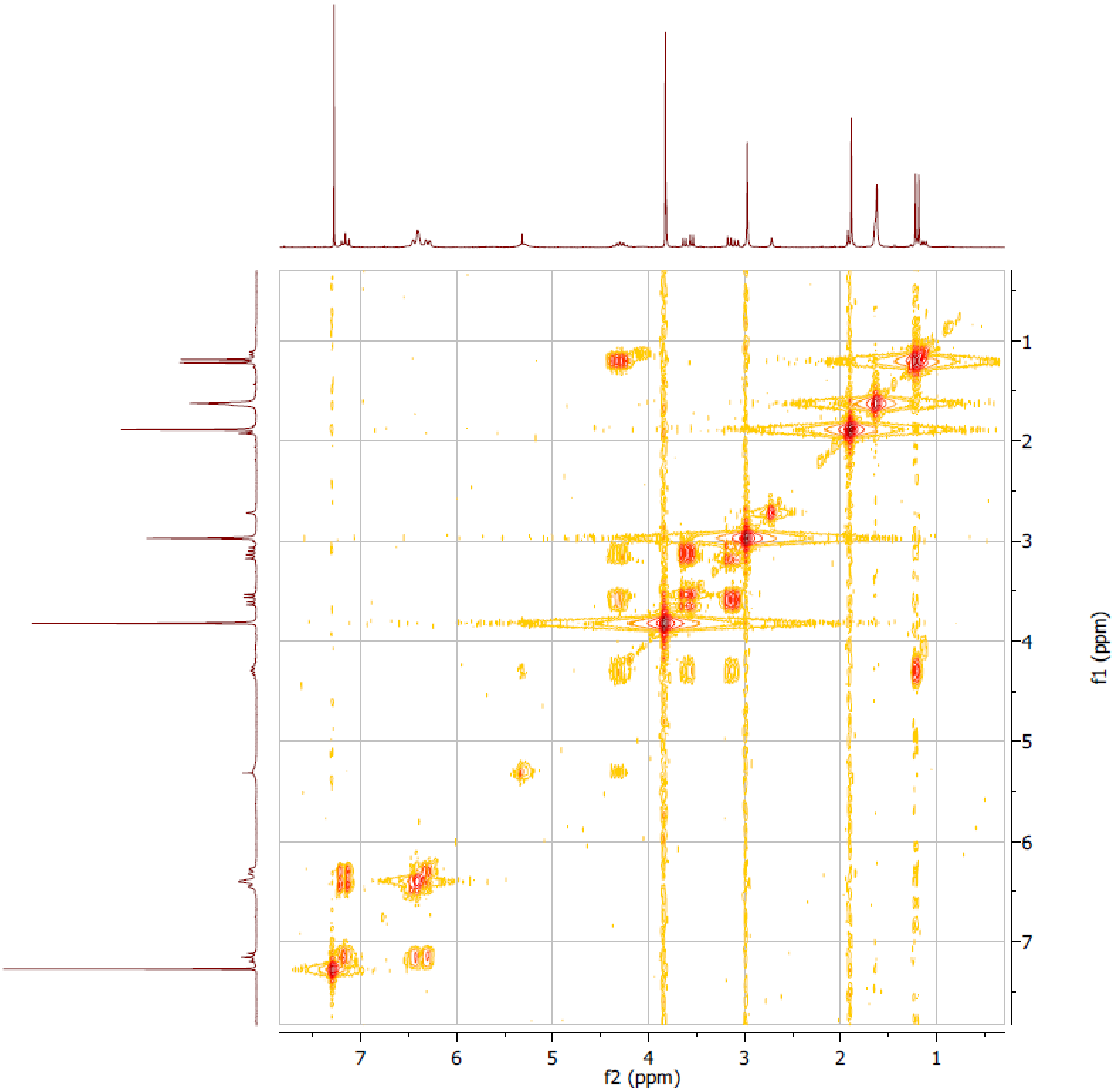

The observed NH-CHα correlation and the absence of a coupling between CH_2 protons and amidic NH is in agreement with the proposed structure.

Figure S14. Chiral HPLC of compound **6**.

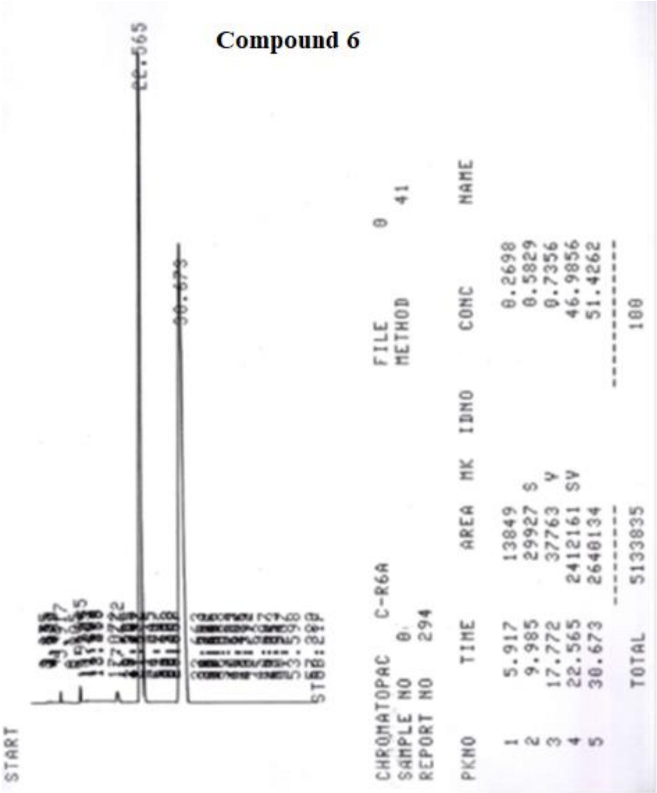

Figure S15. Chiral HPLC of compound **(*R*)-6**.

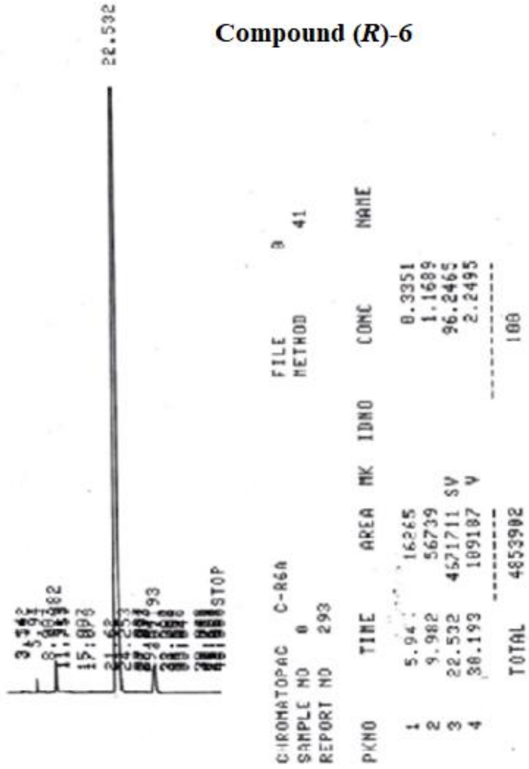

Figure S16. Chiral HPLC of compound **(*S*)-6**.

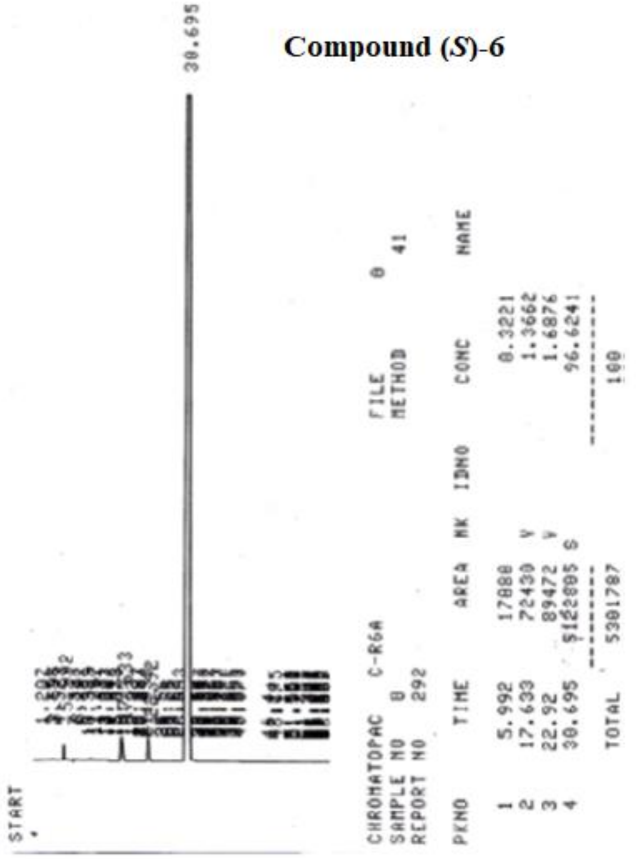

Figure S17. Chiral HPLC of compound **10**.

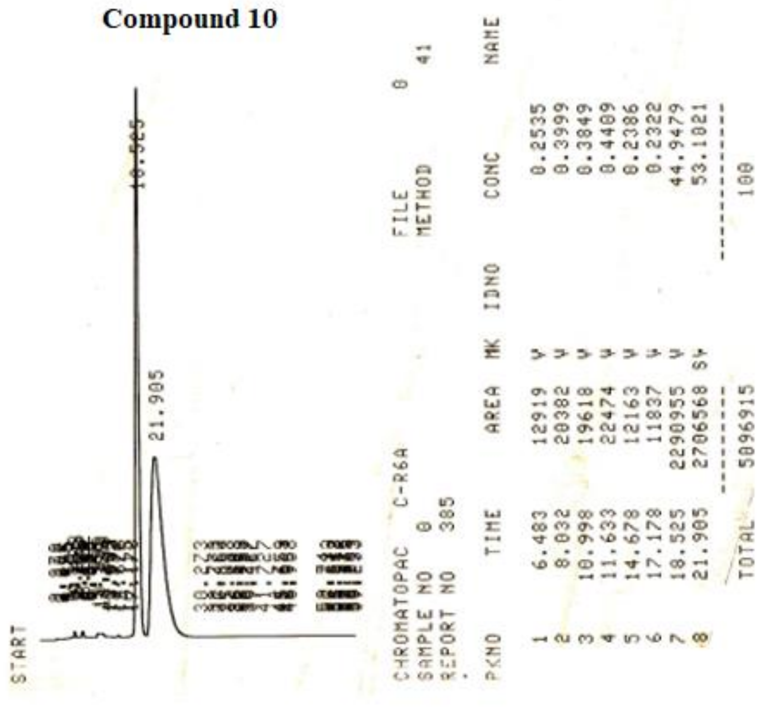

Figure S18. Chiral HPLC of compound **(*R*)-10**.

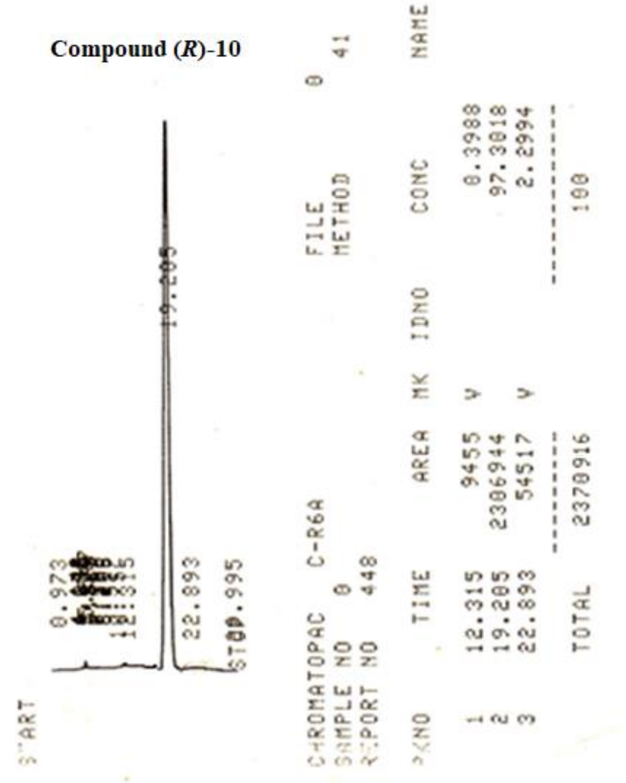

Figure S19. Chiral HPLC of compound **(*S*)-10**.

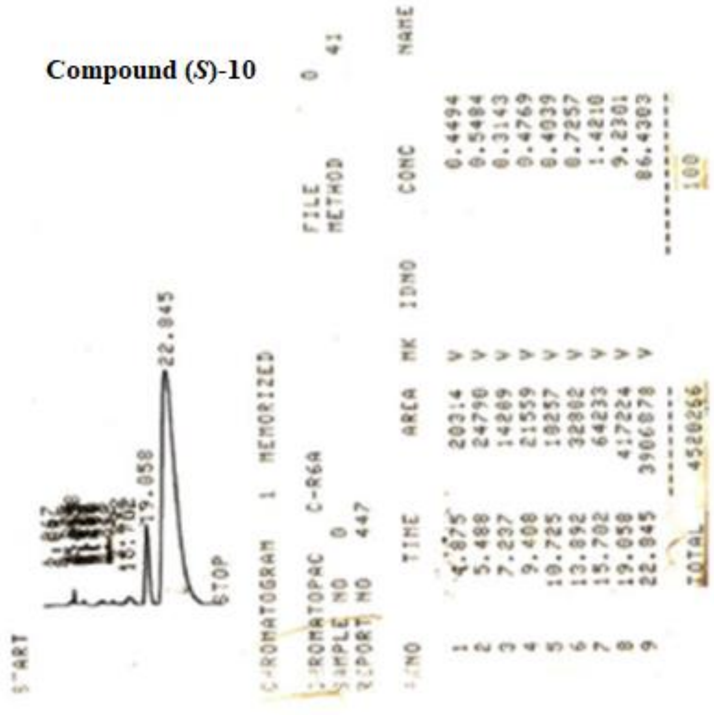

Figure S20. Free-energy surfaces calculated from the MD simulation of **(*S*)-10** in POPC (left) and in TIP3P water molecules (right). Dihedrals values assumed in the minimized docking pose are shown as a black diamond.

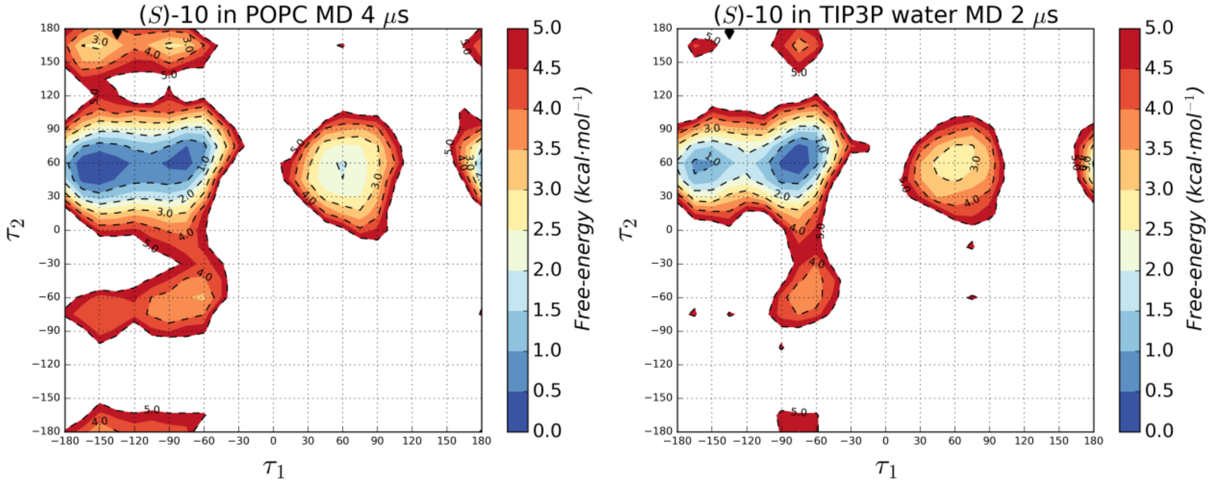

Protocol S1. Equilibration protocol for MD simulations of MT_2 receptor-ligand complexes

The equilibration protocol consists of a prolonged version of the default relaxation procedure implemented in Desmond 5.4 for protein-membrane systems.

1. 50 ps of Brownian dynamics in NVT ensemble with 1 ps timestep (3 ps for long-range electrostatics) for at 10 K with a force constant of 50 $kcal \cdot mol^{-1} \cdot Å^{-2}$ on the solute heavy atoms;
2. 200 ps of Brownian dynamics in NPT ensemble with 1 ps timestep (3 ps for long-range electrostatics) at 100 K with a force constant of 20 $kcal \cdot mol^{-1} \cdot Å^{-2}$ on the solute heavy atoms. A directional restraint on Z-axis was applied to membrane heavy atoms with a force constant of 5 $kcal \cdot mol^{-1} \cdot Å^{-2}$;
3. 500 ps in NPγT ensemble with 1ps timestep (3 ps for long-range electrostatics) at 100 K with a force constant of 10 $kcal \cdot mol^{-1} \cdot Å^{-2}$ on the solute heavy atoms. A directional restraint on Z-axis was applied to membrane heavy atoms with a force constant of 2 $kcal \cdot mol^{-1} \cdot Å^{-2}$;
4. 1.5 ns of gradual heating in NPγT ensemble from 100 K to 300 K with timesteps used during production (2 ps and 6 ps for long-range electrostatics, see main text) and a force constant of 10 $kcal \cdot mol^{-1} \cdot Å^{-2}$ on the solute heavy atoms. A directional restraint on Z-axis was applied to choline heads polar heteroatoms with a force constant of 2 $kcal \cdot mol^{-1} \cdot Å^{-2}$;
5. 4.0 ns in NVT ensemble divided in steps with gradual release of restraints and timesteps as in production:
   - 1.0 ns with restraints on backbone and ligand heavy atoms of 5 $kcal \cdot mol^{-1} \cdot Å^{-2}$;
   - 1.0 ns with restraints on alpha carbons and ligand heavy atoms of 5 $kcal \cdot mol^{-1} \cdot Å^{-2}$;
   - 1.0 ns with restraints on alpha carbons of 2.5 $kcal \cdot mol^{-1} \cdot Å^{-2}$ and ligand heavy atoms of 1 $kcal \cdot mol^{-1} \cdot Å^{-2}$. Henceforth, restraints on cap-termini backbone heavy atoms are kept as in the production phase (see main text);
   - 1.0 ns with further differentiated restraint on the alpha carbons (helices alpha carbons are restrained with a spring constant of 2.5 $kcal \cdot mol^{-1} \cdot Å^{-2}$, while for the other alpha carbons it is reduced to 0.1 $kcal \cdot mol^{-1} \cdot Å^{-2}$) and ligand heavy atoms restrained with a force constant of 0.1 $kcal \cdot mol^{-1} \cdot Å^{-2}$;
6. 1.0 ns in NPγT ensemble with timesteps and restraints as in the production phase.

Figure S21. Time-evolution of the free energy surface calculated from the MD simulation of **(*S*)-10** in explicit chloroform.

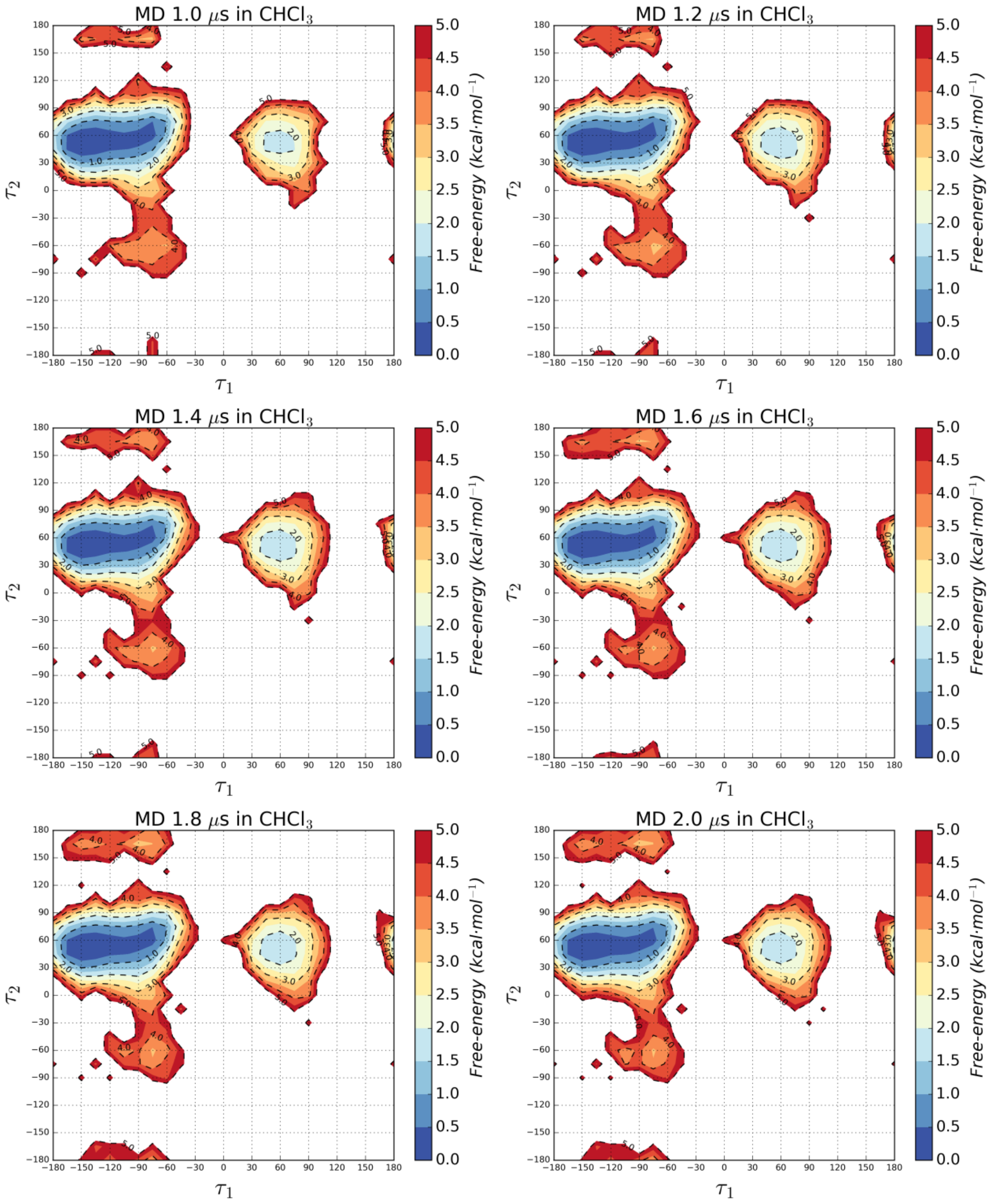

Supplement: Supplementary file 1 [file molecules-25-04057-s001.pdf]
